# CoAID-DEEP: An Optimized Intelligent Framework for Automated Detecting COVID-19 Misleading Information on Twitter

**DOI:** 10.1109/ACCESS.2021.3058066

**Published:** 2021-02-09

**Authors:** Diaa Salama Abdelminaam, Fatma Helmy Ismail, Mohamed Taha, Ahmed Taha, Essam H. Houssein, Ayman Nabil

**Affiliations:** 1 Faculty of Computers and Artificial IntelligenceBenha University 68816 Benha 13511 Egypt; 2 Faculty of Computer ScienceMisr International University 68894 Cairo 11341 Egypt; 3 Faculty of Computers and InformationMinia University 68843 Minia 61519 Egypt

**Keywords:** Fake news, COVID-19, misleading information, pandemic, social media, deep learning

## Abstract

COVID-19 has affected all peoples’ lives. Though COVID-19 is on the rising, the existence of misinformation about the virus also grows in parallel. Additionally, the spread of misinformation has created confusion among people, caused disturbances in society, and even led to deaths. Social media is central to our daily lives. The Internet has become a significant source of knowledge. Owing to the widespread damage caused by fake news, it is important to build computerized systems to detect fake news. The paper proposes an updated deep neural network for identification of false news. The deep learning techniques are The Modified-LSTM (one to three layers) and The Modified GRU (one to three layers). In particular, we carry out investigations of a large dataset of tweets passing on data with respect to COVID-19. In our study, we separate the dubious claims into two categories: true and false. We compare the performance of the various algorithms in terms of prediction accuracy. The six machine learning techniques are decision trees, logistic regression, k nearest neighbors, random forests, support vector machines, and naïve Bayes (NB). The parameters of deep learning techniques are optimized using Keras-tuner. Four Benchmark datasets were used. Two feature extraction methods were used (TF-ID with N-gram) to extract essential features from the four benchmark datasets for the baseline machine learning model and word embedding feature extraction method for the proposed deep neural network methods. The results obtained with the proposed framework reveal high accuracy in detecting Fake and non-Fake tweets containing COVID-19 information. These results demonstrate significant improvement as compared to the existing state of art results of baseline machine learning models. In our approach, we classify the data into two categories: fake or nonfake. We compare the execution of the proposed approaches with Six machine learning procedures. The six machine learning procedures are Decision Tree (DT), Logistic Regression (LR), K Nearest Neighbor (KNN), Random Forest (RF), Support Vector Machine (SVM), and Naive Bayes (NB). The parameters of deep learning techniques are optimized using Keras-tuner. Four Benchmark datasets were used. Two feature extraction methods were used (TF-ID with N-gram) to extract essential features from the four benchmark datasets for the baseline machine learning model and word embedding feature extraction method for the proposed deep neural network methods. The results obtained with the proposed framework reveal high accuracy in detecting Fake and non-Fake tweets containing COVID-19 information. These results demonstrate significant improvement as compared to the existing state of art results of baseline machine learning models.

## I. Introduction

COVID-19 is rumored to be caused by a new SARS-CoV, which first appeared in China in December 2019 and soon spread. The Zika virus outbreak was declared a public health emergency of international significance on January 30, 2020, and the virus was named COVID-19 in March of the same year [1]. According to WHO, as of May 6, 2020, more than 3.5 million cases of COVID-19 have been reported to the World Health Organization. The most common symptoms of CVID-19 infection include cough, trouble breathing, fever, sore throat, and an inability to taste or smell [2].

While in the midst of the COVID-19 scenario, complexities relating to COVID-19 have risen and triggered significant social disturbances. On the other hand, fraudulent purveyors of the COVID-19 commodity have caused many people to suffer. For example, an Arizona man was dead and his wife was hospitalized after the couple ingested a form of Chloroquine to prevent COVID-19. On the other hand, poor contact is adversely affecting social order [1].

Misinformation is one type of the many forms of misinformation such as rumors, misleading content on the internet, and fake news. Many studies have described fake news as “news articles that are intentionally written to mislead or misinform readers, but can be verified as false by means of other sources” Fake news is due to its exposure to public polarization and quarrelling [3], [4].

Various examples are seen in both the 2016 US [5] presidential campaign and the 2019 Indian airstrike in Balakot. Accurately distinguishing between genuine news and fake news will be needed for building intelligent AIs. Social networking stages, including Facebook, Twitter, etc., are struggling to cope with the amount of misinformation that is being shared on these sites.

Fake news can usually be divided into three distinct categories false stories, large scale hoaxes, and satirical fabrications [6]. The aim of the fabricated interviews and malicious intent category is to expose fakes that thrive on social media. Large scale hoaxes seem as if they are reality but are just a lie. The final category are satirical news intended to amuse users and are frequently disguised as real news by authors. The objectivity of news sites is the most critical aspect in assessing their reliability. Malicious web sites adopt the domains of popular, trustworthy websites. Fake news websites can be used to survey readers and create a data collection for research. Unfortunately, false news may easily be identified on trustworthy websites by error. To gather real and false news stories, both honest and fake articles must be collected. Humans are important to detect the accuracy of the news. Alternative verification approaches are not the only means of validating news. crowdsourcing and computational fact checking models are used to annotate dubious news and to provide fact-checking information [7].

A benchmark dataset of fake news has not been agreed until now because of the trouble of collecting fake news and the ambiguity in providing a clear definition [7]. However, some authors created a dataset from the statements collected from social media such as LIAR [8]. Some authors altered Wikipedia sentences to produce statements and provide evidence for or against such claims in Wikipedia articles [9].

Another fake news dataset is collected from Facebook and Twitter [10]–[13]. Finally, the complete dataset is provided in [14], where the authors provided a dataset that contains information about the content and the social context of the news. Researches on fake news make a difference between content features and context features. The content features of fake news are linguistic features. The context features of fake news are the surrounding information such as user’s characteristics and social network-based features [7].

Therefore, there is a high degree of risk publishing fake news over social media. The big truth of news is the need of the hour, and it’s something that we must fix.

Recently, there are many examples for fake news in France and the USA such as fake news about the Presidential candidates during the France and US presidential election, which was shared over thousands of times, and spread quickly. The following examples of fake news that spread quickly and how content broadcasts over social media

• The first example of fake news is titled by the French presidential campaign commercial, sponsored by Saudi Arabia. This is a paper published on February 24, 2017 that argues that French presidential candidate Emmanuel Macron (a centrist candidate) was sponsored by Saudi Arabia (30% of Macron’s campaign funded by Saudi Arabia during France presidential election.). The characteristic indicated that The story is fabricated. A fake site was created to mimic the real site of Le Soir, and to spread false information. The story on Facebook has received over 10,000 likes, shares, and comments.
• The second example of Fake News, Titled by Hillary Clinton, deletes Hillary Clinton from Twitter. This fake news adversely affects how the US presidential elections turn out. punctuation like commas, apostrophes, quotations, question marks and more are omitted, to decrease the model’s computational cost and increase its efficiency.
• The third example of fake news (a tweet circulated over 1,700 times), titled by Marine Le Pen, mocked the “Masha and the Bear” cartoon because the little girl in the story wears a veil. The paper was released on February 26, 2017 and reported that French presidential candidate and National Front chief Marine Le Pen mocked a children”s cartoon, Masha and the Bear, because Masha wore a “veil.” The truth revealed that Marine Le Pen did not tweet that - a short video of the image is doctored. Secretnews.fr published an article discussing the subject in 2014.
• One of four False News stories, The French state, will spend 100 million euros purchasing hotels on housing migrants. It is announced in the news on March 10th 2017 that the Council of Europe Development Bank (CEB) will lend the French state 100 million euros to buy hotels to house asylum seekers. The truth revealed that the claim was misleading and two independent news stories had been conflated and altered to exaggerate the assistance that was given to asylum seekers. The connection has been shared more than 10,000 times on Facebook. stemming is replacing the suffix with a root word to reduce the number of word types in the results. For example, “Making,” will be represented by the word “Made,” and “Maker” will be represented by the word “make.”
• Finally, consider a real tweet by US President Trump. He has ordered a funding freeze on the World Health Organization, accusing it of practicing “blackout” over the spread of the Coronavirus. He found fault with the WHO for not being able to tackle the emerging COVID-19 crisis, which caused the deaths of thousands. The World Health Organization had given misleading information gathered from China, which had spread the virus across its territory. “He also considered that the international organization” Most publications declined to offer the same standard of transparency as this one.

More and more, false allegations have been made on social media that harm the reputations of politicians. Some recent examples have suggested that lawmakers and elected officials indulge in bad conduct but the truth is that the claims are unfounded. Some outlets describe politicians as heroes for tasks they did not achieve. Either way, fake news adversely affects public confidence in the media. Furthermore, tall tales can affect people’s opinions.

The previous examples contribute to the issue of people not knowing because the consumer is misinformed. The negative influence of social media misinformation has a widespread negative impact on society. When people spread false facts, they negatively affect people’s emotions. We made an attempt to model the problem in our proposed model.

The paper contributions of the proposed techniques can be summarized as follows:

1) We have the first initiative to apply deep learning techniques to the COVID-19 dataset to detect false news.
2) We proposed a novel Fake News Detection system on social media platforms for COVID-19 dataset and others using Modified Deep Neural Network methods.
3) We conduct a systematic experiment using various state-of-the-art machine learning algorithms to determine the efficiency of the proposed deep learning algorithms.
4) In Fake News Detection, the proposed algorithm achieved 98.57% accuracy on the best dataset.

The remainder of this paper is organized as follows: the related work is presented in section 2. The proposed methodology is introduced in section 3. The experiment results and discussion are discussed in section 4 and section 5, respectively. Finally, conclusions are presented in section 6.

## II. Related Work

This section introduces different machine learning techniques to detect fake news in general. The first subsection is related to COVID-19 fake news detection since it is the most recent topic.

### A. COVID-19 Fake News Detection

Since the appearance of the first COVID-19 case on December 31, the World Health Organization (WHO) declared it as a pandemic emergency. Social media news and tweets contain information or misinformation about COVID-19. Ordinary people become more anxious to read more to know how to protect themselves. The authors in [25] analyzed the sources of COVID-19 misinformation. Their analysis revealed that most of the misinformation about COVID-19 are fabricated from true information rather than invented. Detecting fake news about COVID-19 attracted data scientists. The authors in [22] applied 10 machine learning algorithms, with 7 feature extraction techniques to detect whether the corpus of news is fake or real. They tested their proposed classifier on 3,047,255 COVID-19 related tweets. The best performance measures are achieved by NN, DT, and LR classifiers. In [26], the authors extracted the textual features of COVID-19 tweets beside user and network features. They proposed mBERT (multilingual Bidirectional Encoder Representations from Transformers) which is a deep neural network approach. mBERT achieved the highest performance measures compared with traditional machine learning techniques such as SVM, RF and a multilayer perceptron. In [20], the authors applied two pipelined pre-trained deep learning natural language frameworks named BERT and ALBERT. They used a public dataset on COVID-19 that contains more than 5000 COVID-19 false claims. Their proposed model yielded the best performance results.

NLP researchers have been working on developing algorithms for the detection of online COVID-19 related disinformation. To develop any algorithm, we require a corpus. So members of the NLP community created the various fake news datasets: FakeCovid [15], ReCOVery [16], CoAID [1], and CMU-MisCOV19 [17]. Yichuan Li *et al.* [18] developed multi-dimensional and multilingual MM-COVID corpora, which covers six languages. Mabrook *et al.* [19] created a large Twitter dataset related to COVID-19 misinformation. And authors developed an ensemble-stacking model with six machine learning algorithms on the created dataset for detecting misinformation.

Elhadad *et al.* [22] constructed a voting ensemble machine learning classifier for fake news detection that uses seven feature extraction techniques and ten machine learning models. Tamanna *et al.* [21] used the COVIDLIES dataset to detect the misinformation by retrieving the misconceptions relevant to the Twitter posts. For COVID-19 fake news detection and fact-checking, Rutvik *et al.* [20] proposed a two-stage transformer model. The first model retrieves the most relevant facts about COVID-19 by using a novel fact-checking algorithm, and the second model, by computing the textual entailment, verifies the level of truth.

also Fake news can be found in form of “fake cures” such as in [23], [24] that point out it influences the decision-making process in medicine

Adapting all these classical and hybrid related work techniques, we developed a COVID-19 fake news detection system in this paper.

### B. Fake News Detection Algorithms

Detecting fake news becomes one of the most critical tasks of artificial intelligence scientists. There are two main approaches for detection: the machine learning approach and the Deep Learning approach.

1) **Machine learning approach for fake news detection:** Deception has been studied and defined as the creation of a false conclusion by transmitting a false message. In their study, [27] analyzed a set of linguistic features and investigated three classifiers. SVM achieved the highest precision, recall, and F-measure. However, linguistic features and visual features are commonly used in SVM approaches such as [27]–[31]. In [32], authors distinguished between fake news, satire news, and real ones by introducing a set of distinguishing features such as the titles. In [33], both content and context-based features were used to detect fake news using a Decision Tree(DT). However, Random Forest (RF) and Decision Tree (DT) were applied using user characteristics in [34], [35] to detect the trustworthiness of users writing the news. Additional features like topic models based features are used in [33]. In [29], the authors defined some linguistic cues of deception and applied Random Forest (RF) to detect fake news. Logistic Regression (LR) has shown competitive performance in detecting fake news in [12], [29], [36].
2) **Deep learning approach for fake news detection:** Deep learning classifiers have become popular in recent years. The approach of deep learning is efficient in terms of extracting relevant features [37]. Recurrent neural networks (RRNs) and, in particular, LSTM is efficient in modeling sequential data [12]. In [38], the authors proposed different RNN architectures, namely tanh-RNN, LSTM, and Gated Recurrent Unit (The Modified GRU), and The Modified GRU achieved the best performance. In [39], LSTM has been fed by a mix of content and context-based features of news, and it achieved good accuracy in detecting fake news. CNN’s are a class of neural networks that gain popularity in the NLP field [40]. In [41], both RNN and CNN are used to detect false news and show a better performance than the performance of baselines. In [42], the authors used LSTM and hybrid LSTM-CNN architectures. The simplest LSTM showed the best performance. In [8], a hybrid model of RNNs and CNNs was used by the authors where the text information is encoded via CNN, and LSTM encodes the metadata of the author. This hybrid model outperformed the baseline model. Table 2 represents a summary of related work

**TABLE 1:** Comparison Between Different Fake News Detection for COVID-19

Ref	Authors	Title	method	Year
[15]	S. Kishore, et al.	FakeCovid(–)A Multilingual Cross-domain Fact Check News Dataset for COVID-19	BERT based classifier without fine-tuning COVID-19 (76%)	2020
[16]	Zhou, et al.	ReCOVery: A Multimodal Repository for COVID-19 News Credibility Research	SAFE Neural Network(83%)	2020
[1]	L.Cui, and D.Lee	CoAID: COVID-19 Healthcare Misinformation Dataset	Deep Learning (89%)	2020
[17]	S.Memon, and K.Carley	Characterizing covid-19 misinformation communities using a novel twitter dataset	Sociolinguistic Analysis	2020
[18]	Y. Li, et al.	MM-COVID: A Multilingual and Multidimensional Data Repository for CombatingCOVID-19 Fake New	Deep Learning (90%)	2020
[19]	E.J. M.Al-Rakhami, and A.Al-Amri	Lies Kill, Facts Save: Detecting COVID-19 Misinformation in Twitter	Ensemble-model(SVM+RF(97.8%))	2020
[20]	R. Vijjali, et al.	Two stage transformer model for covid-19 fake news detection and fact checking	BERT (94.4%)	2020
[21]	T.Hossain, et al.	COVIDLIES: Detecting COVID-19 Misinformation on Social Media	BERT	2020
[22]	M. M.Elhadad, et al.	COVIDLIES: Detecting Misleading Information on COVID-19	ensemble models (BERT, ALBERT, and XLNET)	2020
[23]	V. L.Rundo, et al.	Recent advances of HCI in decision-making tasks for optimized clinical workflows and precision medicine	Gathering dataset only	2020
[24]	A.Ghenai, and Y.Mejova	Fake cures: user-centric modeling of health misinformation in social media	Gathering Dataset only	2018

**TABLE 2:** Comparison Between Different Fake News Frameworks

Ref	Authors	Title	method	features	Year
[38]	L. Zhou, et al.	An exploratory study into deception detection in text-based computer-mediated communication	Logistic regression	content	2003
[36]	C. Castillo, et al.	Information credibility on twitter	Decision tree	Content and context	2011
[27]	H. Zhang, et al.	An improving deception detection method in computer- mediated communication	SVM	Content-based features	2012
[28]	S. Afroz, et al.	Detecting hoaxes, frauds, and deception in writing style online	SVM	Content-based features	2012
[41]	K. Cho, et al.	Learning phrase representations using RNN encoder-decoder for statistical machine translation	Deep learning	Content and context	2014
[29]	E.J. Briscoe, et al.	Cues to deception in social media communications	SVM	Content- based features	2014
[35]	J. Ito, et al.	Assessment of tweet credibility with LDA features	Random forest	Content and context	2015
[30]	V. Perez-Rosas, R. Mihalcea	Experiments in open domain deception detection,	SVM	Content- based features	2015
[37]	M. Hardalov, et al.	In search of credible news	Logistic regression	content	2016
[31]	V. Rubin, et al.	Fake news or truth? Using satirical cues to detect potentially misleading news	SVM	Content- based features	2016
[32]	B.D. Horne, S. Adali	This just in: fake news packs a lot in the title, uses more straightforward, repetitive content in the text body, more similar to satire than real news	SVM	Content-based features	2017
[12]	E. Tacchini, et al.	Some like it hoax: automated fake news detection in social networks	Logistic regression	content	2017
[8]	W.Y. Wang	Liar, liar pants on fire: a new benchmark dataset for fake news detection	ensemble	Content and context	2017
[43]	S. Volkova, et al.	Separating facts from fiction: linguistic models to classify suspiciously and trusted news posts on twitter	RNN and CNN	Content and context	2017
[42]	N. Ruchansky, et al.	a hybrid deep model for fake news detection	Modified LSTM	Content and context	2017
[8]	W.Y. Wang	Liar, liar pants on fire: a new benchmark dataset for fake news detection	Deep learning	Content and context	2017
[44]	O. Ajao, et al.	Fake news identification on twitter with hybrid CNN and RNN models	RNN and CNN	Content and context	2018

## III. Methodology

**FIGURE 1.**
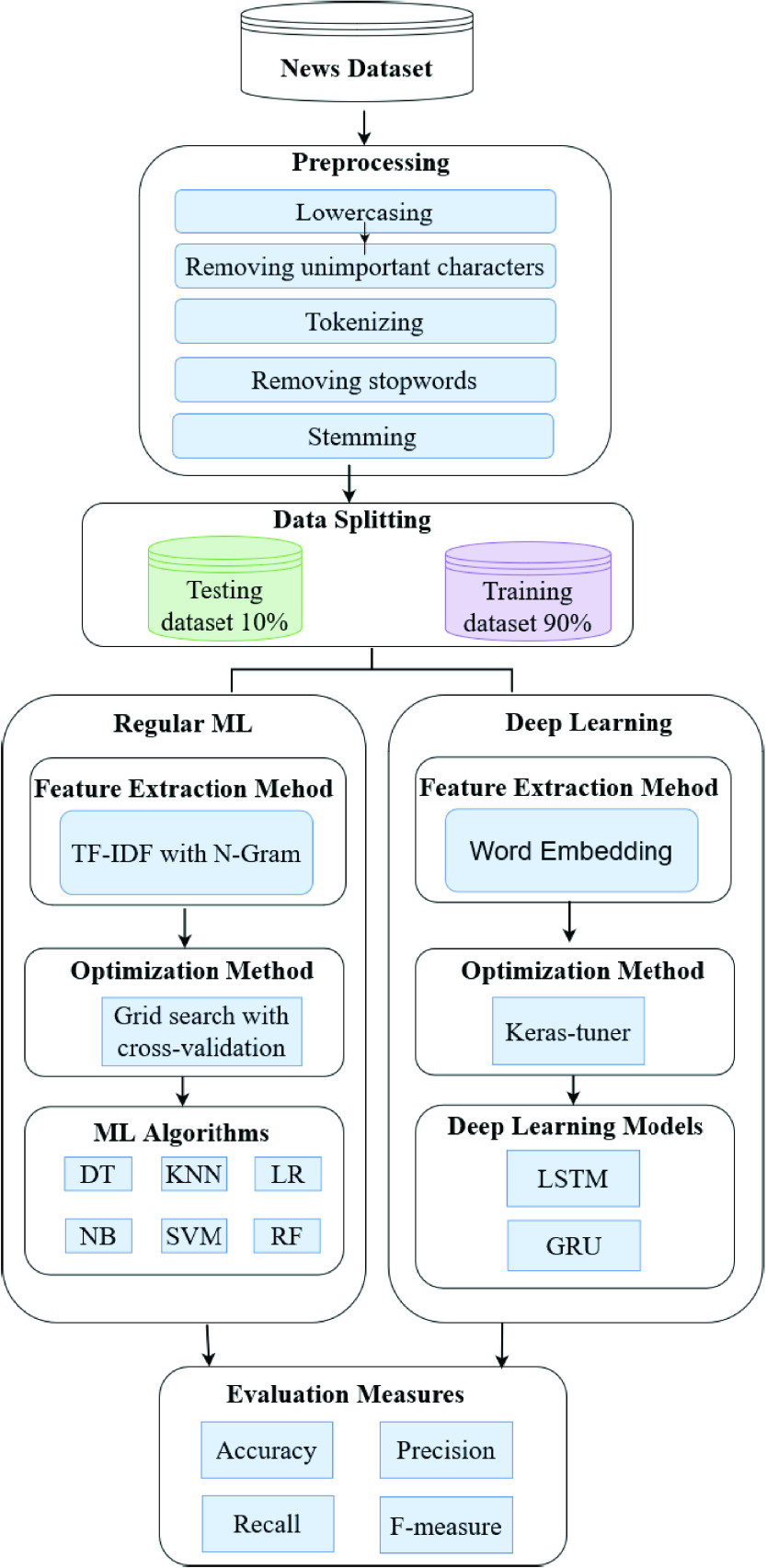
The proposed Framework for Automated Detecting COVID-19 Misleading information onTwitter.

The proposed system of fake news detection consists of two main categories, as shown in Figure 1. The first category uses regular machine learning algorithms, and the second category by using deep neural networks. The first category detects fake news using six baseline traditional machine learning techniques. The machine learning techniques are decision tree (DT), logistic regression (LR), K-nearest neighbor (KNN), random forest (RF), support vector machine (SVM), and Naive Bayes (NB). The second category detects fake news using two proposed deep learning techniques. The two deep learning techniques are Modified LSTM (one to three layers) and Modified GRU (one to three layers). **In the first step (preprocessing step)** for the two categories; includes removing unimportant characters, tokenization, removing stop wording, and stemming. **In the third step**, the feature is extracted using TF-IDF with N-grams for the first category, ML techniques. In contrast, for the second category, i.e., deep learning techniques, the feature is extracted using the word embedding method with Glove to build a word embedding matrix. **In the fourth step**, the parameters of traditional machine learning techniques are optimized using a grid search with stratified cross-validation, while the parameters of deep learning techniques are optimized using a Keras-tuner library. **The performance of each technique** is evaluated by measuring accuracy, precision, recall, and F-Measure. Each step is described in detail in the following subsections.

### A. Data Collection

Experiments were conducted using Four Twitter fake news datasets in different topics. the first topic is CoAID (COVID-19 heAlthcare mIsinformation Dataset) [1]. the secand dataset include disasters [45], PolitiFact [46], gossip cop [46]. The disaster dataset is collected from Kaggle about the topic disaster, while the second and third datasets are related PolitiFact and gossip cop topics collected from FakeNewsNet.

1) **CoAID (COVID-19 heAlthcare mIsinformation Dataset)** is a diverse array of COVID-19 healthcare disinformation, from fake news on blogs and social media, along with the impact that individuals have on such fake news. The data contains 4,251 news mentions, 296,000 user engagements, 926 tweets referencing the COVID-19 and ground truth label.
2) **The disaster dataset [45]** has five features; id, text, location, keyword, and target (see Table [3]). The Disaster dataset has a text of 7613 tweets. Each given tweet is about a real disaster or not labeled as 1 and 0, respectively. In particular, 4342 tweets show a real disaster, while 3271 shows not. In our experiment, we have used two features, which are text and target, as a label, which shows whether a tweet is about a real disaster (1) or not (0).
3) **The PolitiFact dataset PolitiFact [46]** has two files 1) politifact_real.csv, which contains samples related to real news that includes 432 tweets, 2) politifact_fake.csv contains samples related to fake news that includes 618 tweets. We merged politifact_real.csv and politifact_fake.csv files into one file where each tweet belongs to politifact_real labeled as 0 while each tweet belongs to politifact_fake labeled as 1. The final PolitiFact dataset has five features: id, URL, title, tweet-id, and label (see Table ([4]). In our experiment, we used the title to represent the text of the tweet and label features.
4) **The gossip cop dataset [46]** has two files 1) gossip cop_real.csv, which contains sample tweets related to real news that includes 5328 tweets, 2) gossipcop_fake.csv contains sample tweets related to fake news. We selected 5322 tweets from gossipcop_fake.csv. We merged gossipcop_real.csv and gossipcop_fake.csv files into one file where each tweet belongs to gossipcop_real labeled as 0 while each tweet belongs to gossipcop_fake labeled as 1. The final gossip cop dataset has five features, including id, URL, title, tweet-id, and label (see Table [4]). In our experiment, we used the title to represent the text of the tweet and label features.

**TABLE 3:** The Disaster Dataset Description

Features	Description
Id	a unique identifier for each tweet
text	the text of the tweet
location	the location the tweet was sent from (may be blank)
keyword	a particular keyword from the tweet (may be blank)
target	target denotes whether a tweet is about a real disaster (1) or not (0)

**TABLE 4:** The Final Politifact and Final Gossip Cop Datasets Description

Features	Description
Id	Unique identifiers for each news
URL	Url of the article from the web that published that news
title	Title of the news article
tweet_ids	Tweet ids of tweets sharing the news. This field is a list of tweet ids separated by a tab.
label	The label denotes whether a tweet is about a real disaster (1) or not (0)

### B. Data Preprocessing

Data preprocessing is an important phase for any sentiment analysis system, especially for social media content. Twitter data is the popular unstructured datasets collected of information from people entered his/her feelings, opinion, attitudes, products review, emotions, etc. These datasets need to be subjected to certain refinements by performing preprocessing techniques to the next phases of elaboration, i.e., applying ML/DL techniques. The basic cleaning operations within preprocessing techniques used in this work are removing unimportant characters, stop-word removal, tokenization, a lower casing, sentence segmentation, and punctuation removal. They will help us to reduce the size of actual data by removing the irrelevant information that exists in the data and then to achieve better performances. In our study, the preprocessing involves a series of techniques which are listed in the following steps:

• **Lower casing:** simply, it one of the basic cleaning operations to convert a word to lower cases such as NLP -> nlp.
• **Removing unimportant data:** the punctuation like commas, apostrophes, quotes, question marks, and more which do not add much value to a natural language model are deleted.
• **Tokenization:** It is the key aspect of working with text data to separate a piece of text into smaller units called tokens. The tokens are including paragraphs and sentences which can be further broken into words. For example, consider this sentence before tokenization: “never give up”, after tokenization it comes ‘never’, ‘give,’ ‘up’.
• **Removal of Stop Word:** a stop word usually refers to the most common words in a language that does not add much meaning to a sentence such as articles, prepositions and conjunctions, and some pronouns. These words are removed from each tweet with the datasets.
• **Stemming:** stemming is removing the suffix from and transform it to its root word to reduce the number of word types or classes in the data. For example, the words “Making,” “Made,” and “Maker” will be reduced to the word “make.”

### C. Data Splitting

In this step, a dataset from 90 percent of the training set and another dataset from 10 percent of the testing set are used. The training set is fed into the ML/DL models to find out what should be done with the data, and the unseen test set is used as a check on the results.

### D. Applying Optimization and Learning Models

In this step, we applied using two Learning models (Regular Machine Learning algorithms, Deep Learning models). Further details about each model are presented as follows:

#### 1) Regular Machine Learning

We applied six machine learning models after two steps. firstly is by applying Machine Learning Feature Extraction Method then optimized the models using Hyperparameters Optimization Methods. Further details about each model are presented as follows:

1) **Machine Learning Feature Extraction Method:** In this step, we have used the TF-IDF feature extraction method with different sizes of N-Gram and 3000 matrix size. N-gram is the simplest model that assigns probabilities to sentences and sequences of words beginning with length n. The value of n can be one (unigram), two (bi-gram), three (tri-gram) and so on. Various unigrams and bigrams can be classified into two groups: character-based, and word-based. A specific set of text characters taken from a phrase. We are using this technique since related terms would have a high proportion of N-grams in common. Typical values for n are 2 or 3; these correspond to bigrams or trigrams, respectively. For example, the word computer results in:
  • the generation of the bigrams *C, CO, OM, MP, PU, UT, TE, ER, R*
  • o and the generation of the trigrams **C, * CO, COM, OMP, MPU, PUT, UTE, TER, ER*, R** Where ‘*’ denotes a padding space. Character-based N-grams are generally used in measuring the similarity of character strings. The Term Frequency-Inverted Document Frequency (TF-IDF) is a well-known feature method to evaluate the importance of a word in a document. According to this work, the TF-IDF method is used to measure the importance of a term within a tweet in the fake news datasets. The key idea of the TF-IDF method is converting the tweets into a Vector Space Model (VSM) and then calculating the importance of the term by counting its frequencies within the tweets. The word-based frequency is counted using different n-grams, including uni-gram, bi-gram, and tri-gram, etc.
2) **Hyperparameters Optimization Methods:** In this step, we have used the hyperparameters optimization techniques to select the best value for each parameter of regular machine learning models, including Grid Search with stratified 10-fold cross-validation described as follows:
  • Grid search is a hyperparameter optimization technique for hyperparameter tuning, which is used to methodically select the best value that achieves the best performances for an ML model. It evaluates ML model for each combination of algorithm parameters specified in a grid and then reports the optimal values of model hyperparameters.
  • K-Fold Cross-Validation is mainly used for hyperparameter tuning by dividing the sample of datasets into a training set to train the model, and a test set to evaluate it. The dataset is split into k equal partitions where k-1 groups are used for training, and the one fold is held for the testing model. This process is repeated k times (i.e., k = 10), including one fold, is used for testing and k-1 folds for the training set. In our experiment, we used k = 10. In the 10-fold CV process, 90% of data were used for the training, and 10% of data were used for testing purposes.
3) **Machine Learning Models:** We have used six regular machine learning algorithms, which are Decision Tree, Random forest, K-Nearest Neighbor, Logistic Regression, Support Vector Machine, and Naive Bayes, to classify news into fake and real news. Further details about each model are presented as follows:
  • **Decision Tree (DT)** [47] are useful supervised Machine learning algorithms that can perform our classification tasks in this paper. It consists of nodes and branches, where the tests on each attribute are represented at the nodes, the outcome of this procedure is represented at the branches, and the class labels are represented at the leaf nodes. The goal is to create a model that classifying the value of a target variable by learning simple decision rules concluded from the data features.
  • **Random forest (RF)** [48], [49] is a supervised machine learning algorithm that uses a collection of decision trees, providing more flexibility, accuracy, and ease of access. This algorithm dominates over decision trees algorithm as decision trees provide low accuracy compared to the random forest algorithm. In simple words, the random forest approach increases the performance of decision trees. It is one of the best algorithms in classification techniques, and we used it in our paper. The goal is to create a model that classifying the value of a target variable by learning simple decision rules concluded from the data features from more than the tree.
  • **K-Nearest Neighbor (KNN)** [50], [51] is a Supervised classification algorithm. It is one of the most straightforward and widely used algorithms which depends on its k value; K specifies the number of neighbors, and its algorithm is as follows:
    • Choose the number K of neighbor.
    • Take the K Nearest Neighbor of a new data point, according to Euclidean Distance. (We can increase or decrease it as you like to get the best accuracy that we needed)
    • Among the K-neighbors, Count the number of data points in each category.
    • Assign the new data point to a category, where you counted the most neighbors
  • **Support vector machine (SVM)** [52], [53] SVM is a supervised learning system used for classification problems with an associated algorithm. In this process, each data object is plotted in n-dimensional space with the value along with the coordinate deciding the value of the item. Classification is achieved by discovering the hyper-plane best dividing the categories. SVMs are capable of performing a non-linear classification by directly translating inputs into high-dimensional feature spaces.
  • **Logistic Regression (LR)** [54] is a Machine Learning algorithm used for classification problems. It is a predictive analysis algorithm and based on the concept of probability. It is based on the sigmoid function where output is the probability (Value of output ranges from 0 to 1), and input can be from-infinity to +infinity. If we need to classify our data into two classes, then if the output probability range is less than 0.5, then our data in the first class (class tag (0)), and if the probability range more than 0.5, then our data in the second class (class tag (1)).
  • **Naive Bayes (NB)** [55] is a probabilistic machine learning model based on Bayes’ theorem. It makes classifications using the Maximum Posterior decision rules in a Bayesian setting.

#### 2) Deep Learning

We applied Two Deep learning models after two steps. firstly is by applying Deep Learning Feature Extraction Method then optimized the models using Hyperparameters Optimization Methods. Further details about each model are presented as follows:

1) **Deep Learning Feature Extraction Method** In this step, we have used word embeddings, which generally converts text data, i.e., words into vectors. It represents every word in an n-dimensional dense vector where similar words will have a similar vector. The more efficient word embeddings techniques which have proven there capability to convert words into vectors are GloVe and Word2Vec. According to this work, GloVe [56] represents the tweets within the fake news datasets into dense vectors, which fed into the deep learning models. The gloVe is an unsupervised learning algorithm for word embeddings, which is used to obtain the vector representations for words. The key idea of the GloVe technique is to discover the closeness of two words, with their separation in a vector space to create vector representations called word embedding vectors. The embedding vectors are created by aggregating global word-word co-occurrence statistics from the datasets and then resulting in the matrix representations, including measuring the closeness of two words in a tweet. We used glove.twitter.27B.zip that includes a different dimension of vectors, which are 25d, 50d, 100d, and 200d vectors. We used 200d vectors to build the embedding matrix.
2) **Hyperparameters Optimization Method:** For hyper-parameters optimization, we have used a Keras-tuner [57] library to pick the optimal set of hyperparameters in hidden layers (Modified LSTM or Modified GRU) and dropout layers. We set different values for different parameters: the number of neurons, reg_rate for l2 regularization technique [58], and the dropout rate for the dropout layers [59]. For this, we have applied the Keras-tuner on the training dataset to select the best parameters, as shown in 5.
3) **Deep neural network:** 2 shows the deep neural network architecture that is used to classify news into fake and real. It consists of a word embedding matrix as input to embedding layer, embedding layer, hidden layers including Long Short-Term Memory (LSTM), or Gated Recurrent Units (GRU), flatten layer [60], and output layer. In the word embedding matrix, the GloVe word embedding technique has been used to calculate word embeddings using a co-occurrence matrix in between words within fake news tweets, which is called the embedding matrix. The embedding matrix is used to represent the tweets into dense vectors. The embedding layer, hidden layers, and output layer are described as follows:
  • **Embedding layers** The embedding layer is implemented in the Keras library [61]. Regarding this work, Keras library is used to initialize the embedding layer to learn an embedding for all of the words in the training dataset. The **Keras** embedding layer has three arguments, including
    a) **input_dim** defines the size of the vocabulary in the dataset.
    b) **output_dim** defines the size of the vector space in which words will be embedded.
    c) **input_length** defines the length of input sequences as defined for any input layer of a Keras model. The embedding layer is configured as follows; **input_dim** equals 20000 because the number of words is 20000, **output_dim equals 200 and because we used 200d vectors of golvetweet and input_length equals 32.**
  • **Hidden Layer** Two different neural network models are used; LSTM or GRU model. For each model, a different number of hidden layers has been applied, including one, two, and three layers. Also, one dropout layer and different numbers of neurons in each hidden layer have been used. The ReLU (Rectified Linear Unit) [62] activation function has been applied for the hidden layers. For each hidden layer, l2 regularization techniques have been used by adopting reg_rate value for l2. Also, we used the dropout layer and the different number of dropout rate.
  • Output Layer The output layer provides the final output of the model where the neural network model classifies the inputs tweets into two categories; real or fake. In particular, the output layer has one neuron, which detected the news within an input tweet in terms of fake or real. In this layer, we used the ADAM optimizer [63] and sigmoid [64] is the activation function.
4) **Recurrent Neural Network (RNN):**
  Problems that cannot be boiled down to a set number of inputs and outputs. Problems in which the device is needed to store and use background information. Hard/impossible to choose the exact meaning of a word There is always new data available that is longer than everything else. A recurrent neural network is useful because it allows for the intermediate values (states) to store information about past inputs for a time that is not set a priori [65]. The RNN repeats the same task for each element in the sequence [66]. The RNN uses the secret state to store the state of each moment, and the state depends on the previous moment and current input. The new secret state effectively capitalizes on the past knowledge. Thus, an RNN can perform this function via dynamic processes. The architecture of an RNN is depicted in Figure 3. Given an input sequence $X=[x_{1},x_{2} \cdotp \cdotp x_{t}\cdotp \cdotp x_{T}]$ of length T, an RNN determines the hidden state ht at the time t of the sequence as in equation (1):
  \begin{equation*} h_{t}=\tanh \left ({W_{h} h_{t-1}+W_{x} x_{t}+b}\right)\tag{1}\end{equation*}
  Despite the great value of RNNs for learning sequential patterns, the gradient descent method is difficult to implement because of the well-known gradient vanishing/explosion problem [67]. To solve these two separate issues, research is being performed on Long Short-Term Memory (LSTM), GRU, etc. Consequently, modified GRU, and modified LSTM are chosen as our methodology.
5) **Long Short-Term Memory network (LSTM):** Long Short-Term Memory network (LSTM) [40], [68] is a deep recurrent neural network which is more reliable than the traditional recurrent neural network when used in tasks with long time lags. The main difference between an RNN and an LSTM is that an RNN has a single tanh layer, while an LSTM has four interactive LSTM layers (see Figure 4). The LSTM memory cell is composed of a memory block and three multiplicative gating units. For the gate, the sigmoidal nature of the function $\sigma $ ranges from 0 to 1 [69], [70].
  LSTM has three of these gates to protect and control the cell state. The three gated are the
  • **Forget gate layer** $f_{t}$, as shown in Figure (6 a). Forget gate layer is used to decide what information throw away from the cell state). Forget gate layer as shown in Equation (2) Output a number is between 0 and 1.
    \begin{equation*} f_{t}=\sigma \left ({W_{f} \cdot \left [{h_{t-1}, x_{t}}\right]+b_{f}}\right)\tag{2}\end{equation*}
  • Then **Add new information** as shown in Figure (6 b) is to decide what new information store in the cell state - **Input gate** layer $i_{t}$ as shown in Equation (3)
    \begin{equation*} i_{t}=\sigma \left ({W_{i} \cdot \left [{h_{t-1}, x_{t}}\right]+b_{i}}\right)\tag{3}\end{equation*}
    then Decides which values we will update as shown in Figure (6 c) (Tanh layer as shown in Equation (4) $\tilde {C}_{\mathrm {t}}$) by creating a vector of new candidate values
    \begin{equation*} \tilde {C}_{t}=\tanh \left ({W_{C} \cdot \left [{h_{t-1}, x_{t}}\right]+b_{C}}\right)\tag{4}\end{equation*}
  • **Update cell state** $C_{t}$, as shown in Figure (6 c): by Forgetting the things we decided to forget earlier $\mathrm {f}_{\mathrm {t}} * \mathrm {C}_{\mathrm {t}-1}$ and Adding information we decide to be added ${\mathrm {i}}_{\mathrm {t}} * \widetilde {\mathrm {C}}_{\mathrm {t}}$ as shown in the following formula (5)
    \begin{equation*} C_{t}=f_{t} * C_{t-1}+i_{t} * \tilde {C}_{t}\tag{5}\end{equation*}
  • **Create Output as shown in Figure (6) (Output gate layer $o_{t}$, Tanh layer)**by Decide what we are going to Output as shown in (5 d)
    • Output gate layer $o_{t}$ as shown in Equation (6): Decides what parts of the cell state we are going to Output
      \begin{equation*} o_{t}=\sigma \left ({W_{o}\left [{h_{t-1}, x_{t}}\right]+b_{o}}\right)\tag{6}\end{equation*}
    • **Tanh layer** $h_{t}$: as shown in Equation (7) Push the values between −1 and +1
      \begin{equation*} h_{t}=o_{t} * \tanh \left ({C_{t}}\right)\tag{7}\end{equation*}
  • **Peephole** as shown in Figure (6 e) to Let the gate layer look at the cell state (entire/ partial) as shown in the following Equations (8)
    \begin{align*} f_{t}=&\sigma \left ({W_{f} \cdot \left [{\boldsymbol {C}_{t-1}, h_{t-1}, x_{t}}\right]+b_{f}}\right) \\ i_{t}=&\sigma \left ({W_{i} \cdot \left [{\boldsymbol {C}_{t-1}, h_{t-1}, x_{t}}\right]+b_{i}}\right) \\ o_{t}=&\sigma \left ({W_{o} \cdot \left [{\boldsymbol {C}_{t}, h_{t-1}, x_{t}}\right]+b_{o}}\right)\tag{8}\end{align*}
    •Coupled forgot and input gates as shown in Figure 6: f Not deciding separately as shown in the following Equations (9)
    \begin{equation*} C_{t}=f_{t} * C_{t-1}+\left ({1-f_{t}}\right) * \tilde {C}_{t}\tag{9}\end{equation*}
    So we can summarise the LSTM into four steps
    • **Step 1:** Forget gate layer
    • **Step 2:** Input gate layer
    • **Step 3:** Combine step 1 and 2
    • **Step 4:** Output the cell state
    Though LSTM uses a certain kind of RNN. LSTM learning techniques are able to learn long term dependencies. Although LSTM cannot learn to fill a wide gap in knowledge, RNNs do not have a gap problem. LSTM minimizes the number of losses. LSTM embraces character sequences of varying lengths, such so that no linguistic features are needed to be extracted [5]. This algorithm is also compact; the update complexity per weight and time step and storage complexity per weight is on the order of O (1)
  • **Concept of the Gated Recurrent Unit (GRU):**
    The GRU has the same structure as either a basic RNN or STM, except the GRU updates the hidden state [71], [72].. The key difference between LSTM and GRU is that LSTM combines forget and input layers into a single “update gate”, combines cell state and hidden state, and is more convenient and common. Instead of explicitly updating the current hidden state with the previous hidden state, GRU uses a reset gate and updates the gate, deciding if the information in the previous hidden state is useful, then retains useful information and removes useless information [73]. Figure (5) shows the architecture of GRU.
    The way GRU updates $h_{t}$ is as follows:
    a) **The reset gate $r_{t}$ and update gate** $z_{t}$ as shown in Equation (10) and in Equation Equation (11)
      \begin{align*} z_{t}=&\sigma \left ({W_{z h} h_{t-1}+W_{z x} x_{t}+b_{z}}\right)\tag{10}\\ r_{t}=&\sigma \left ({W_{r h} h_{t-1}+W_{r x} x_{t}+b_{r}}\right)\tag{11}\end{align*}
      For the gate, $\sigma $ is a logistic sigmoid, The reset gate $rt$, and update gate $zt$ ranging from 0 to 1.
    b) **Candidate hidden state** $\tilde {h}_{t}$:
      \begin{align*} \tilde {h}_{t}=\tanh \left ({W_{\tilde {h} h}\left ({r_{t} * h_{t-1}}\right)+W_{\tilde {h} x} x_{t}+b_{h}}\right) \\\tag{12}\end{align*}
      The candidate hidden state ($\tilde {h}_{t}$) is shown in Equation (12), and it uses the reset gate $r_{t}$ to monitor the inflow of the previous hidden state $h_{t-1}$, which contains past details. If the reset value is set to 0, the previous state will be restored. Hence, the reset gate provides a method to to delete past secret states which are not related to the future; that is, the reset gate decides how much information was forgotten.
    c) **hidden state $h_{t}$:**
      \begin{equation*} h_{t}=z_{t} * h_{t-1}+\left ({1-z_{t}}\right) * \tilde {h}_{t}\tag{13}\end{equation*}
      Hidden state ($h_{t}$) is shown in Equation (13). It updates previous hidden state and the candidate hidden state $h {t-1}$ with an update gate. If the update gate $z_{t}$ is 1, the long-held previous secret state $h_{t}$ can be moved to the current moment. The GRU can handle the probability gradient vanishing problem in the RNN, so it is more suitable for detecting faults in dynamic systems.
6) **Evaluating models** Four statistics are used to determine the consistency of models. TP is True Positive, TN is True Negative, FP is False Positive, and FN is False Negative. Accuracy is shown in Equation (14) and Precision is shown in Equation (15). Recall is shown in Equation (16), and F1-Score is shown in Equation (17)
  • **Accuracy** is a measure of totally correctly identified samples out of all the samples.
    \begin{equation*} {~\text {Accuracy }}=\frac {T P+T N}{T P+T N+F P+F N} \times 100\tag{14}\end{equation*}
  • **Precision and Recall** The measure of the ability of the model to accurately identified the occurrence of a positive class instance is determined by recall
    \begin{align*} {~\text {Precision }}=&\frac {T P}{T P+F P}\tag{15}\\ \text {Recall}=&\frac {T P}{T P+F N}\tag{16}\end{align*}
  • **F1-Score** The harmonic mean of Precision and Recall
    \begin{equation*} F 1-{~\text {Score }}=\frac {2 * {~\text {Precision }} * {~\text {Recall }}}{{~\text {Precision }}+{~\text {Recall }}}\tag{17}\end{equation*}

**FIGURE 2.**
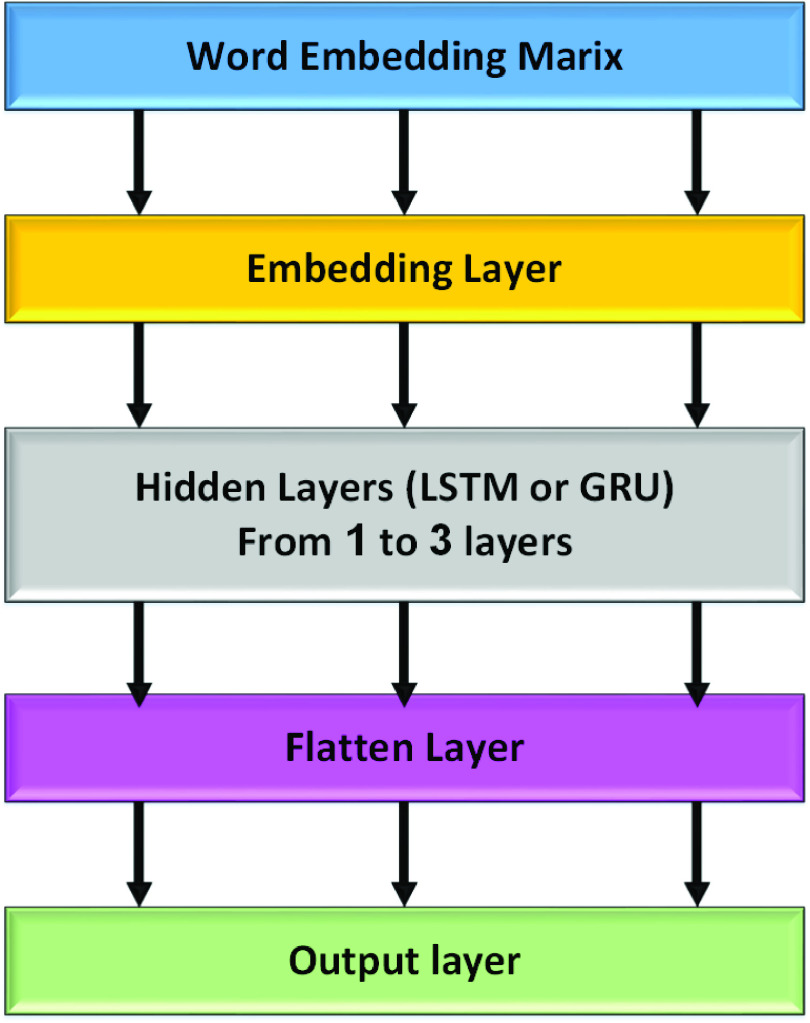
Deep neural network architecture.

**FIGURE 3.**
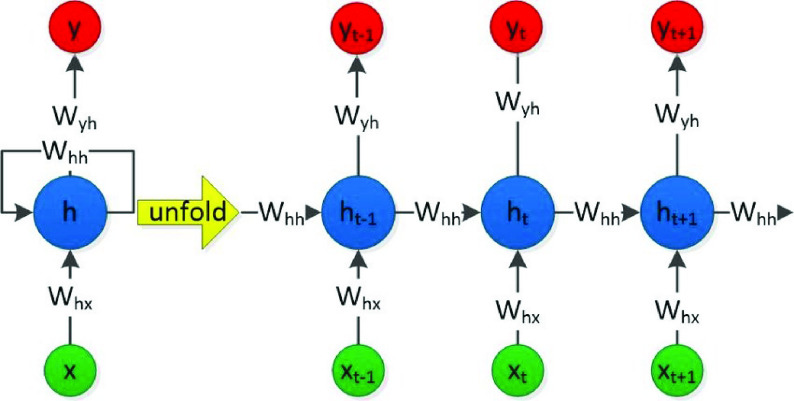
RNN architecture.

**FIGURE 4.**
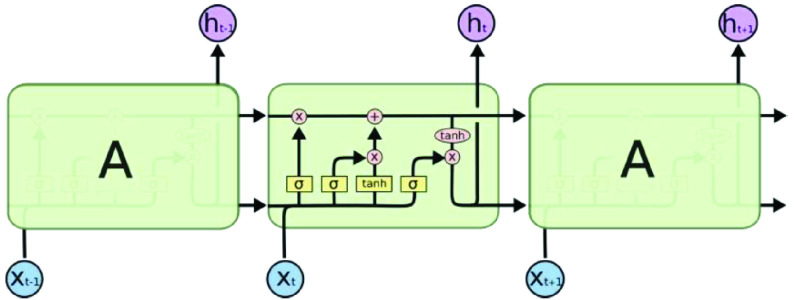
LSTM architecture.

**FIGURE 5.**
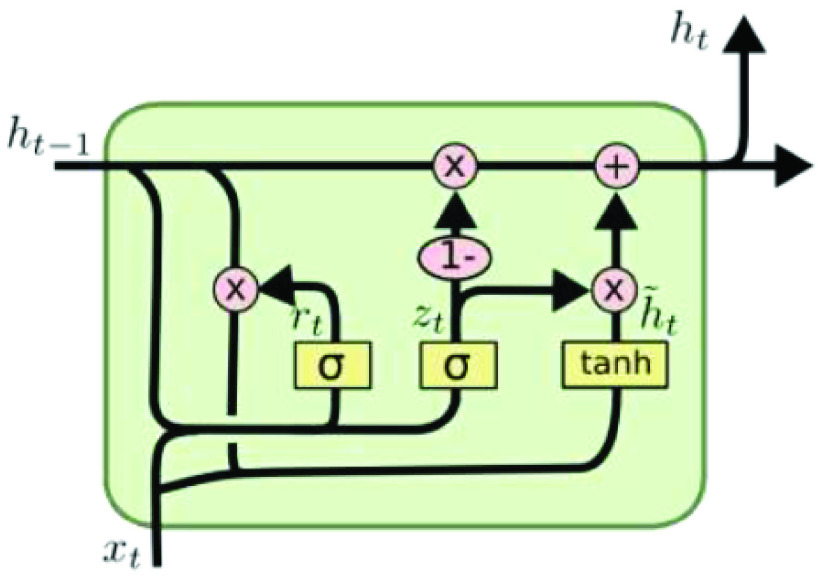
GRU architecture.

**FIGURE 6.**
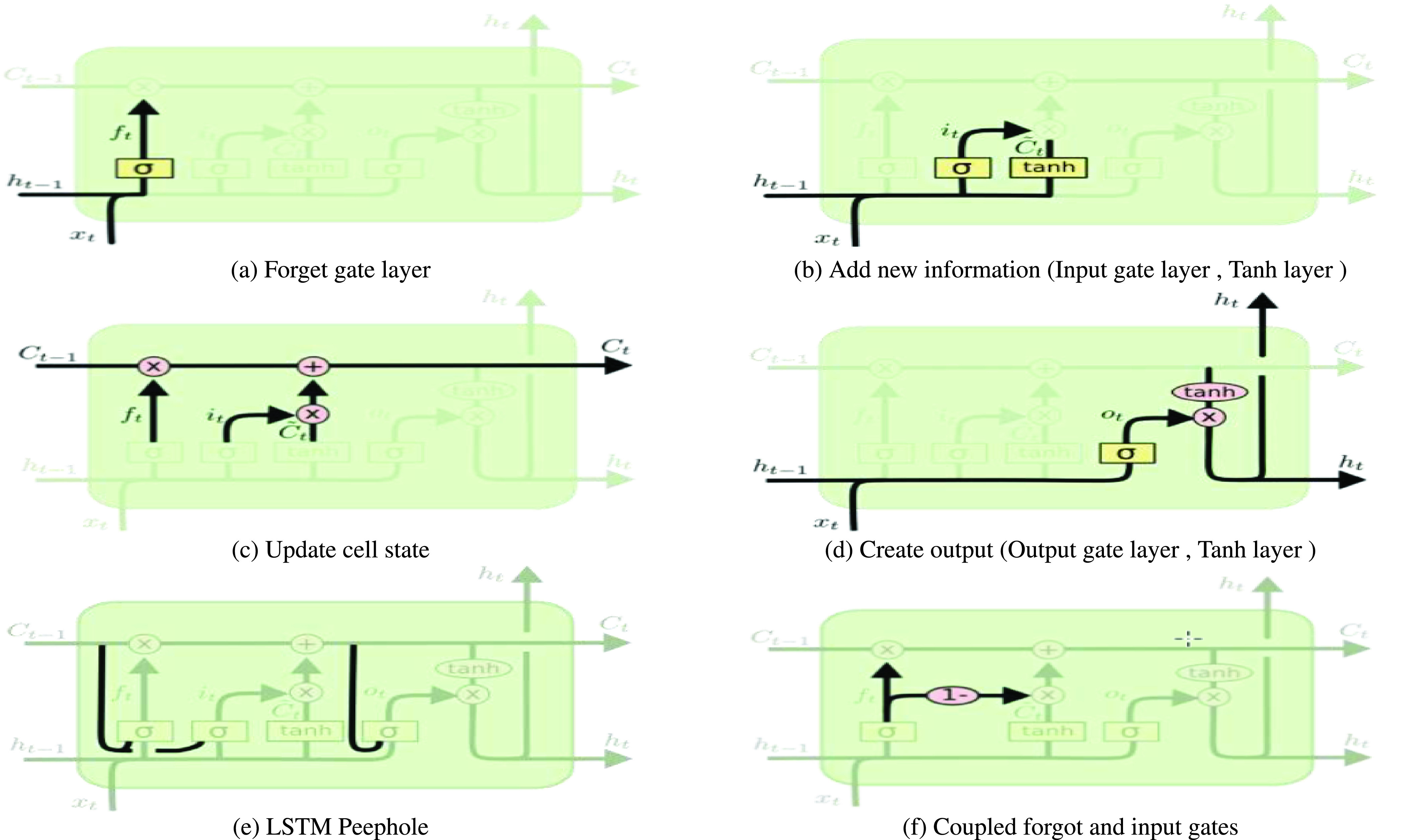
LSTM layers Steps.

## IV. Experimental Results and Discussion

In this section, the results of applying six machine learning models (DT, LR, KNN, RF, SVM, NB) and Two deep learning models (Modified LSTM, Modified GRU), including cross-validation results and testing results, are described. Each machine learning model performance is discussed using four sizes of TF-IDF feature extraction, including uni-gram, bi-gram, tri-gram, four-gram, and one matrix size; 3000. In this section, the experimental evaluation of the proposed models is presented, starting by describing the experiment setup.

### A. Experimental Setup

The ML and DL models are trained with 90% of the dataset and then are tested with the 10% testing data. The six ML classifiers are implemented using the sci-kit-learn package in Python 3. The DL models are implemented using TensorFlow and Keras package in Python 3.

### B. Experiment I (COAID (COVID-19 Healthcare Misinformation Dataset))

#### 1) Hyperparameter Tuning

The method of selecting hyperparameters is a vital part of any deep learning solution. Deep learning algorithms all have specifically defined parameters that govern various aspects of them. Hyperparameters are the variables that are set prior to using a particular algorithm on a specific set of data. The best numbers depend on each task and each context-dependent dataset. The best values of parameters for the two deep learning models (Modified LSTM, Modified GRU) are shown in **Table [6]**.

**TABLE 5:** Hyperparameters Configurations Selected by Keras-Tuner

Parameter	The value
Dropout rate	within the range of 0.1 rates to 0.5 rate
The number of neurons	within the range of 10 neurons to 200 neurons
regularization l2	0.01, 0.05, 0.1,.2,.3,.4,.5

**TABLE 6:** The Best Values Hyperparameter for the COVID 19 Dataset for Modified LSTM and Modified GRU

DL Models	Neurons	Dropout	reg_rate
Modified LSTM one layer	110	0.6	0.4
Modified LSTM two layers	[290,470]	[0.4,0.5]	[0.01,02]
Modified LSTM three layers	[90, 350, 130]	[0.3,0.2,0.3]	[0.05, 0.01,02]
Modified GRU one layer	450	0.4	0.1
Modified GRU two layers	[290,170]	[0.9,0.2]	[0.01,04]
Modified GRU three layers	[190, 350, 110]	[0.7,0.9,0.3]	[0.01,0.4,0.3]

#### 2) Cross-Validation Result for the COVID 19 Dataset

We evaluate the performance of six machine learning models over a collection of datasets and show that, on average, the models predict 10 times better than random guessing (3000).

• the results of cross-validation are shown in **Table [7]** with DT, LR, KNN, RF, SVM, NB, respectively using 3000 matrix size have obtained higher output for all TF-IDF feature extraction methods, including uni-gram, bi-gram, tri-gram and four-gram for machine learning models and word embedding feature extraction method for deep learning models. The Uni-gram model using known text obtained the highest efficiency (accuracy of 96.22%, the precision of 95.19%, recall of 96.23% and F1-score 95.35%). Similar to KNN, the use of four-grams resulted in the best results (accuracy of 95.87%, the precision of 94.35%, recall of 95.87% and F1-score 94.66%). The RF model using bi-gram yielded the highest efficiency (accuracy of 96.63%, precision of 96.41%, recall of 96.63% and F1-score 96.52%). As to LR, the highest performances have been obtained using bi (twice) gram (accuracy of 96.38%, the precision of 95.73%, recall of 96.38% and F1-score 95.17%). As the results show that the logistic regression model with two coefficients obtained the best efficiency (accuracy of 96.64%, the precision of 96.45%, recall of 96.64% and F1-score 95.53%). The NB model using four-gram resulting in the highest efficiency (accuracy of 96.24%, the precision of 95.67%, recall of 96.24% and F1-score 94.73%).
• Regarding deep learning models, as shown in **Table [8]**: Modified LSTM, two layers, which have the highest efficiency (accuracy of 98.57%, the precision of 98.82%, recall of 99.71%, and F1-score 99.26%). Modified GRU one layer, achieved the best efficiency (accuracy of 98.33%, the precision of 98.67%, recall of 99.62%, and F1-score 99.14%).

**TABLE 7:** The Performance of ML for Cross-Validation Results (COVID 19 Dataset)

Feature extraction method	ML Models	Matrix Size	Measure Performance Methods			
			Accuracy	Precision	Recall	F1-score
Unigram	DT	3000	96.22± 0.1	95.19± 0.15	96.23± 0.1	95.35± 0.11
Bi-Gram			96.14± 0.1	95.08± 0.2	96.18± 0.1	95.26± 0.13
Tri-Gram			96.11± 0.13	94.98± 0.18	96.09± 0.12	95.21± 0.14
Four-gram			96.1± 0.12	95.0± 0.23	96.1± 0.12	95.21± 0.15
Unigram	KNN	3000	95.68± 0.33	93.99± 0.29	95.68± 0.33	94.49± 0.2
Bi-Gram			95.82± 0.28	94.26± 0.36	95.82± 0.28	94.63± 0.21
Tri-Gram			95.85± 0.26	94.31± 0.37	95.85± 0.26	94.64± 0.19
Four-gram			95.87± 0.24	94.35± 0.33	95.87± 0.24	94.66± 0.18
Unigram	RF	3000	96.57± 0.07	96.22± 0.15	96.57± 0.08	95.47± 0.14
Bi-Gram			96.63± 0.08	96.41± 0.14	96.63± 0.08	95.52± 0.17
Tri-Gram			96.53± 0.06	96.17± 0.15	96.55± 0.09	95.41± 0.13
Four-gram			96.54± 0.07	96.03± 0.16	96.54± 0.07	95.44± 0.14
Unigram	LR	3000	96.28± 0.04	95.4± 0.17	96.28± 0.04	95.01± 0.07
Bi-Gram			96.38± 0.07	95.73± 0.21	96.38± 0.07	95.17± 0.11
Tri-Gram			96.35± 0.06	95.66± 0.21	96.35± 0.06	95.1± 0.09
Four-gram			96.36± 0.07	95.72± 0.25	96.36± 0.07	95.09± 0.1
Unigram	SVM	3000	96.63± 0.08	96.42± 0.15	96.63± 0.08	95.54± 0.16
Bi-Gram			96.64± 0.09	96.45± 0.15	96.64± 0.09	95.53± 0.18
Tri-Gram			96.63± 0.08	96.41± 0.14	96.63± 0.08	95.52± 0.17
Four-gram			96.62± 0.08	96.41± 0.14	96.62± 0.08	95.51± 0.17
Unigram	NB	3000	96.17± 0.02	95.64± 0.25	96.17± 0.02	94.55± 0.04
Bi-Gram			96.24± 0.04	95.68± 0.22	96.24± 0.04	94.72± 0.08
Tri-Gram			96.24± 0.05	95.68± 0.23	96.24± 0.05	94.72± 0.09
Four-gram			96.24± 0.04	95.67± 0.21	96.24± 0.04	94.73± 0.08

**TABLE 8:** The Performance of Cross-Validation for Deep Neural Networks (COVID 19 Dataset)

DL Models	Measure Performance Methods			
	Accuracy	Precision	Recall	F1-score
Modified LSTM one layer	98.37± 0.1	98.59± 0.15	99.74± 0.14	99.16± 0.05
Modified LSTM two layers	98.57± 0.14	98.82± 0.18	99.71± 0.14	99.26± 0.07
Modified LSTM three layers	97.72± 0.12	97.98± 0.14	99.68± 0.18	98.82± 0.06
Modified GRU one layer	98.33± 0.16	98.67± 0.16	99.62± 0.19	99.14± 0.08
Modified GRU two layers	98.15± 0.15	98.46± 0.22	99.63± 0.24	99.04± 0.08
Modified GRU three layers	97.62± 0.1	97.95± 0.17	99.61± 0.2	98.77± 0.05

#### 3) Testing Results for the COVID 19 Dataset

In this section, we discuss the generalization performance of the six machine learning models using the unseen test dataset with matrix sizes (3000).

• As the results shown in **Table [9]** described the testing performance of machine learning models including DT, LR, KNN, RF, SVM, NB, respectively.
  . the uni-gram model using the DT technique obtained the highest efficiency (accuracy of 95.0%, the precision of 93.71%, recall of 95.0% and F1-score 94.26%). The KNN model using uni-gram obtained the highest accuracy (accuracy of 95.66%, the precision of 92.16%, recall of 95.66% and F1-score 93.81%). The RF bi-gram model was the most effective; it had the greatest efficiency (accuracy of 96.35%, the precision of 96.07%, recall of 96.35% and F1-score 95.0%). The LR model that used four grams of text achieved the best overall efficiency (accuracy of 95.82%, precision of 94.24%, recall of 95.82% and F1-score 94.59%). An unigram SVM achieved the best overall efficiency (accuracy of 96.36%, the precision of 96.18%, recall of 96.38% and F1-score 95.05%). Using the tri-gram NB model, obtained the highest score (accuracy of 96.13%, the precision of 95.74%, recall of 96.13% and F1-score 94.5%).
• Regarding deep learning models, as shown in **Table [10]**: Modified LSTM, two layers got the highest results (accuracy of 98.6%, the precision of 98.55%, recall of 98.6% and F1-score 98.5%). ‘Modified GRU, a one layer system, achieved the best efficiency (accuracy of 98.29%, the precision of 98.24%, recall of 98.29% and F1-score 98.21%).

**TABLE 9:** The Performance of ML for Testing Result (COVID 19 Dataset)

Feature extraction method	ML Models	Matrix Size	Measure Performance Methods			
			Accuracy	Precision	Recall	F1-score
Unigram	DT	3000	95.0	93.71	95.0	94.26
Bi-Gram			94.94	93.64	94.94	94.2
Tri-Gram			94.82	93.49	94.82	94.08
Four-gram			94.82	93.53	94.82	94.1
Unigram	KNN	3000	95.66	92.16	95.66	93.81
Bi-Gram			95.54	92.21	95.54	93.77
Tri-Gram			95.56	92.23	95.56	93.78
Four-gram			95.59	92.36	95.59	93.81
Unigram	RF	3000	96.1	94.95	96.1	94.83
Bi-Gram			96.35	96.07	96.35	95.0
Tri-Gram			96.08	94.89	96.08	94.8
Four-gram			95.97	94.6	95.97	94.73
Unigram	LR	3000	94.73	93.75	94.73	94.19
Bi-Gram			94.83	93.67	94.83	94.18
Tri-Gram			94.77	93.57	94.77	94.1
Four-gram			95.82	94.24	95.82	94.59
Unigram	SVM	3000	96.38	96.18	96.38	95.05
Bi-Gram			96.36	96.09	96.36	95.02
Tri-Gram			96.35	96.07	96.35	95.0
Four-gram			96.35	96.07	96.35	95.0
Unigram	NB	3000	96.06	95.29	96.06	94.37
Bi-Gram			96.12	95.54	96.12	94.51
Tri-Gram			96.13	95.74	96.13	94.5
Four-gram			96.12	95.54	96.12	94.51

**TABLE 10:** The Performance of Testing Results of Deep Neural Networks (COVID 19 Dataset)

DL Models	Measure Performance Methods			
	Accuracy	Precision	Recall	F1-score
Modified LSTM one layer	98.41	98.33	98.41	98.28
Modified LSTM two layers	98.6	98.55	98.6	98.5
Modified LSTM three layers	97.77	97.63	97.77	97.46
Modified GRU one layer	98.29	98.24	98.29	98.21
Modified GRU two layers	98.24	98.17	98.24	98.06
Modified GRU three layers	97.79	97.63	97.79	97.47

### C. Experiment II: Fake News for (Disasters Dataset)

[45]

#### 1) Hyperparameter Tuning

The best values parameters for two deep learning models (Modified LSTM, Modified GRU) are shown in Table [11].

**TABLE 11:** The Best Values Hyperparameter of the Disasters Dataset for Modified LSTM and Modified GRU

Models	neurons	Dropout	reg_rate
Modified LSTM one layer	290	0.5	0.01
Modified LSTM two layers	[350,430]	[0.9,03]	[0.1,0.4]
Modified LSTM three layers	[190, 210,370]	[0.4,0.2,0.1]	[0.1,0.3,0.3]
Modified GRU one layer	410	0.4	0.1
Modified GRU two layers	[470,70]	[0.7,0.3]	[0.5,0.01]
Modified GRU three layers	[430, 370, 110]	[0.4,0.3,0.2]	[0.1,0.2,0.4]

#### 2) Cross-Validation Results for Disasters Dataset

We experimentally demonstrate the performance of the 10-fold CV results of the six machine learning models over the used Disasters Dataset with one matrix sizes (3000).

• As the results of cross-validation on the Disasters Dataset shown in Table [12] using DT, LR, KNN, RF, SVM, NB, respectively, the dataset using 3000 matrix size have obtained higher performance for all TF-IDF feature extraction methods, including uni-gram, bi-gram, tri-gram and four-gram for machine learning models and word embedding feature extraction method for deep learning models. We attribute this behavior to the larger number of words within the matrix. In particular, when the number of words is a bit larger, the weighting metric becomes more significant, and this improves the machine learning and deep learning models’ performance. However, the machine learning models’ performance using 3000 matrix sizes are varied based on the model and the feature extraction method. For example, Table [12], the DT model using uni-gram achieved the best efficiency (accuracy of 75.3%, the precision of 75.58%, recall of 75.31, and F1-score 74.83%). Similarly to the KNN model, the best efficiency has been obtained using uni-gram (accuracy of 77.03%, the precision of 78.44%, recall of 77.03% and F1-score 76.08%). Similar to the RF model, the best efficiency has been obtained using uni-gram achieved the best efficiency (accuracy of 78.64%, the precision of 79.41%, recall of 78.64% and F1-score 78.08%). Regarding LR, the highest performances have been obtained using bi-gram achieved the best efficiency (accuracy of 79.91%, the precision of 80.28%, recall of 79.91% and F1-score 79.53). The SVM model has obtained the best performance using Bi-gram (accuracy of 80.08%, the precision of 80.69%, recall of 80.08, and F1-score 79.61%). The NB has recorded the highest improvements using uni-gram and 3000 matrix size (accuracy of 79.65%, the precision of 79.96%, recall of 79.65 and F1-score 79.28%).
• $\bullet $ Regarding deep learning models, show in Table [13], the dataset using the word embedding feature extraction method for deep learning models obtained higher performance for all layers, including one layer, two layers, and three layers. The Modified LSTM one layer achieved the best efficiency (accuracy of 82.68%, the precision of 85.86%, recall of 72.13and F1-score 78.14%). Modified GRU one layer achieved the best efficiency (accuracy of 80.37%, the precision of 83.37%, recall of 70.93%, and F1-score 76.21%).

**TABLE 12:** The Performance of ML for Cross-Validation Results (Disasters Dataset)

Feature extraction method	ML Models	Matrix Size	Measure Performance Methods			
			Accuracy	Precision	Recall	F1-score
Unigram	DT	3000	75.3± 1.26	75.58± 1.48	75.31± 1.39	74.83± 1.36
Bi-Gram			74.93± 1.31	75.1± 1.45	74.8± 1.39	74.32± 1.54
Tri-Gram			74.85± 1.42	74.98± 1.48	74.88± 1.44	74.24± 1.51
Four-gram			75.13± 1.36	75.39± 1.4	75.16± 1.32	74.61± 1.35
Unigram	KNN	3000	77.03± 1.3	78.44± 1.37	77.03± 1.3	76.08± 1.42
Bi-Gram			76.28± 1.46	78.41± 1.51	76.28± 1.46	74.99± 1.65
Tri-Gram			75.96± 1.44	78.15± 1.48	75.96± 1.44	74.62± 1.64
Four-gram			75.28± 1.43	76.94± 1.49	75.28± 1.43	74.05± 1.62
Unigram	RF	3000	78.64± 1.4	79.41± 1.34	78.64± 1.38	78.08± 1.4
Bi-Gram			78.4± 1.35	79.32± 1.39	78.35± 1.3	77.6± 1.51
Tri-Gram			78.44± 1.38	78.98± 1.36	78.44± 1.37	78.05± 1.39
Four-gram			78.51± 1.35	79.33± 1.37	78.54± 1.38	77.94± 1.46
Unigram	LR	3000	79.8± 1.26	80.13± 1.26	79.8± 1.26	79.43± 1.32
Bi-Gram			79.91± 1.3	80.28± 1.3	79.91± 1.3	79.53± 1.37
Tri-Gram			79.64± 1.26	79.93± 1.27	79.64± 1.26	79.28± 1.31
Four-gram			79.66± 1.3	79.93± 1.32	79.66± 1.3	79.32± 1.35
Unigram	SVM	3000	79.98± 1.24	80.67± 1.25	79.98± 1.24	79.48± 1.32
Bi-Gram			80.08± 1.27	80.69± 1.27	80.08± 1.27	79.61± 1.35
Tri-Gram			79.88± 1.27	80.48± 1.26	79.88± 1.27	79.41± 1.34
Four-gram			79.34± 1.35	79.73± 1.36	79.34± 1.35	78.92± 1.41
Unigram	NB	3000	79.65± 1.18	79.96± 1.22	79.65± 1.18	79.28± 1.22
Bi-Gram			79.48± 1.15	80.17± 1.21	79.48± 1.15	78.95± 1.21
Tri-Gram			79.21± 1.2	80.14± 1.25	79.21± 1.2	78.59± 1.28
Four-gram			79.22± 1.29	79.82± 1.34	79.22± 1.29	78.72± 1.35

**TABLE 13:** The Performance of Results Deep Neural Networks for Cross-Validation for the Disasters Dataset

Models	Measure Performance Methods			
	Accuracy	Precision	Recall	F1-score
Modified LSTM one layer	82.68± 0.77	85.86± 2.13	72.13± 2.71	78.14± 1.24
Modified LSTM two layers	80.66± 0.78	82.64± 2.71	71.36± 3.77	75.95± 1.68
Modified LSTM three layers	80.92± 0.86	82.65± 3.17	70.99± 4.24	75.75± 1.84
Modified GRU one layer	80.37± 0.61	83.37± 2.68	70.93± 3.44	76.21± 1.27
Modified GRU two layers	79.85± 0.69	79.97± 3.05	70.71± 3.54	74.07± 1.17
Modified GRU three layers	80.23± 0.59	80.53± 2.94	70.75± 3.73	75.02± 1.05

#### 3) Testing Results for Disasters Dataset

• As the results of testing on the Disasters Dataset shown in Table [14] using DT, LR, KNN, RF, SVM, NB, respectively. The DT model using uni-gram achieved the best efficiency (accuracy of 68.42%, the precision of 68.38%, recall of 68.42%, and F1-score 67.44%). Similar to the KNN model, the best efficiency has been obtained using uni-gram (accuracy of 66.68%, the precision of 73.85%, recall of 66.68 and F1-score 61.16%). Similar to the RF model, the best efficiency has been obtained using uni-gram (accuracy of 71.48%, the precision of 75.08%, recall of 71.48, and F1-score 69.04%). Regarding LR, the highest performances have been obtained using Four-gram (accuracy of 73.7%, the precision of 76.65%, recall of 73.7 and F1-score 71.77%). The SVM model has obtained the best performance using bi-gram obtained (accuracy of 72.9%, the precision of 76.65%, recall of 72.9 and F1-score 70.6%). The NB has recorded the highest improvements using Four-gram achieved the best efficiency (accuracy of 76.2%, the precision of 76.26%, recall of 76.2 and F1-score 75.81%).
• $\bullet $ Regarding deep learning models, show in Table [15], the dataset using the word embedding Regarding deep learning models. The Modified LSTM, one layer achieved the best efficiency (accuracy of 86.74%, the precision of 86.98%, recall of 86.74% and F1-score 86.6%). The Modified GRU one layer achieved the best efficiency (accuracy of 81.44%, the precision of 81.68%, recall of 81.44 % and F1-score 81.81%).

**TABLE 14:** The Performance of ML for Testing Results (Disasters Dataset)

Feature extraction method	ML Models	Matrix Size	Measure Performance Methods			
			Accuracy	Precision	Recall	F1-score
Unigram	DT	3000	68.42	68.38	68.42	67.44
Bi-Gram			68.36	68.24	68.36	67.43
Tri-Gram			67.58	67.41	67.58	66.64
Four-gram			68.4	68.3	68.4	67.4
Unigram	KNN	3000	66.68	73.85	66.68	61.16
Bi-Gram			65.93	75.25	65.93	59.4
Tri-Gram			63.9	74.32	63.9	55.84
Four-gram			64.13	73.5	64.13	56.52
Unigram	RF	3000	71.48	75.08	71.48	69.04
Bi-Gram			71.4	74.34	71.4	68.87
Tri-Gram			71.24	74.86	71.24	68.55
Four-gram			70.98	74.52	70.98	68.27
Unigram	LR	3000	73.35	76.72	73.35	71.21
Bi-Gram			73.69	76.74	73.69	71.72
Tri-Gram			73.49	76.6	73.49	71.47
Four-gram			73.7	76.65	73.7	71.77
Unigram	SVM	3000	72.11	76.64	72.11	69.33
Bi-Gram			72.9	76.65	72.9	70.6
Tri-Gram			72.81	76.43	72.81	70.48
Four-gram			73.82	75.09	73.82	72.57
Unigram	NB	3000	75.51	76.99	75.51	74.37
Bi-Gram			75.37	76.26	75.37	74.06
Tri-Gram			75.12	77.17	75.12	73.71
Four-gram			76.2	76.26	76.2	75.81

**TABLE 15:** The Performance of Testing Results of Deep Neural Networks on the Disasters Dataset

DL Models	Accuracy	Precision	Recall	F1-score
Modified LSTM one layer	86.74	86.98	86.74	86.6
Modified LSTM two layers	85.13	85.38	85.13	84.97
Modified LSTM three layers	81.81	82.13	81.81	81.56
Modified GRU one layer	81.44	81.68	81.44	81.81
Modified GRU two layers	81.18	81.38	81.18	80.95
Modified GRU three layers	81.05	81.31	81.05	80.21

### D. Case III: (The Politifact Dataset) Fact-Checking the U.S Political News

[46]

#### 1) Hyperparameter Tuning

The best values parameters for two deep learning models (Modified LSTM, Modified GRU) are shown in Table [16].

**TABLE 16:** The Best Values Hyperparameter of the PolitiFact Dataset for Modified LSTM and Modified GRU

DL Models	Neurons	Dropout	reg_rate
Modified LSTM one layer	270	0.3	0.4
Modified LSTM two layers	[330,370]	[0.1,0.4]	[0.01,02]
Modified LSTM three layers	[270, 330, 130]	[0.3,0.8,0.3]	[0.05, 0.01,02]
Modified GRU one layer	430	0.4	0.1
Modified GRU two layers	[290,170]	[0.4,0.3]	[0.01,04]
Modified GRU three layers	[230, 150, 130]	[0.4,0.5,0.2]	[0.01,0.4,0.3]

#### 2) Cross-Validation Result for the Politifact Dataset

We experimentally demonstrate the performance of the 10-fold CV results of the six machine learning models over the used dataset with one matrix sizes (3000).

• As the results of cross-validation on the PolitiFact dataset shown in Table [17] using DT, LR, KNN, RF, SVM, NB using 3000 matrix size have obtained higher performance for all TF-IDF feature extraction methods, including uni-gram, bi-gram, tri-gram and four-gram for machine learning models and word embedding feature extraction method for deep learning models. The DT model using four-gram achieved the best efficiency (accuracy of 76.8%, the precision of 76.41%, recall of 76.8% and F1-score 76.73%). Similar to the KNN model, using uni-gram achieved the best efficiency (accuracy of 76.67%, the precision of 79.96%, recall of 78.72% and F1-score 75.02%). Similar to the RF model using bi-gram achieved the best efficiency (accuracy of 78.15%, precision of 79.42%, recall of 79.02% and F1-score 77.74%). Regarding LR, the highest performances have been obtained using tri-gram (accuracy of 81.91%, the precision of 81.94%, recall of 81.91and F1-score 81.55%). The SVM model using bi-gram achieved the best efficiency (accuracy of 81.91%, the precision of 82.23%, recall of 82.02% and F1-score 81.69%). The NB model using uni-gram achieved the best efficiency (accuracy of 82.92%, the precision of 83.01%, recall of 82.92% and F1-score 82.82%).
• Regarding deep learning models, as shown in Table [18]: The Modified LSTM, two layers achieved the best efficiency (accuracy of 94.21%, the precision of 96.15%, recall of 88.0%, and F1-score 91.76%). The Modified GRU one layer achieved the best efficiency (accuracy of 88.8%, the precision of 87.85%, recall of 78.17%, and F1-score 81.85%).

**TABLE 17:** The Performance of ML for Cross-Validation Results (The PolitiFact Dataset)

Feature extraction method	ML Models	Matrix Size	Measure Performance Methods			
			Accuracy	Precision	Recall	F1-score
Unigram	DT	3000	75.76± 4.02	75.61± 4.58	76.02± 4.0	74.75± 4.51
Bi-Gram			76.13± 4.0	76.61± 3.96	76.01± 3.91	75.15± 3.69
Tri-Gram			76.65± 3.69	75.65± 3.91	76.61± 3.4	75.79± 4.0
Four-gram			76.8± 3.59	76.41± 4.36	76.8± 3.9	76.73± 3.61
Unigram	KNN	3000	76.67± 3.57	79.96± 3.78	78.72± 3.71	75.02± 3.43
Bi-Gram			74.59± 3.54	74.90± 4.21	74.59± 3.54	72.77± 3.99
Tri-Gram			75.02± 3.43	71.47± 4.68	76.67± 3.57	73.91± 4.65
Four-gram			74.72± 3.34	79.22± 3.77	74.72± 3.34	70.82± 4.65
Unigram	RF	3000	77.62± 3.54	78.92± 3.99	77.54± 3.3	75.64± 4.11
Bi-Gram			78.15± 3.23	79.42± 3.94	79.02± 3.85	77.74± 3.25
Tri-Gram			77.62± 3.4	75.79± 3.64	78.14± 3.45	76.26± 4.09
Four-gram			77.96± 3.52	79.22± 3.73	78.06± 3.41	76.26± 3.99
Unigram	LR	3000	80.85± 3.52	81.14± 3.71	80.85± 3.52	80.12± 3.8
Bi-Gram			81.42± 3.33	80.85± 3.55	81.59± 3.47	81.42± 3.33
Tri-Gram			81.91± 3.61	81.94± 3.7	81.91± 3.61	81.55± 3.78
Four-gram			81.46± 3.28	81.7± 3.46	81.46± 3.28	80.84± 3.51
Unigram	SVM	3000	80.56± 3.39	80.87± 3.62	80.56± 3.39	79.83± 3.64
Bi-Gram			81.91± 3.56	82.23± 3.77	82.02± 3.62	81.69± 3.44
Tri-Gram			81.69± 3.44	81.02± 3.67	81.91± 3.56	81.27± 3.79
Four-gram			81.03± 3.5	81.45± 3.76	81.03± 3.5	80.26± 3.78
Unigram	NB	3000	82.92± 3.68	83.01± 3.77	82.92± 3.68	82.82± 3.73
Bi-Gram			82.32± 3.67	82.66± 3.67	82.32± 3.67	82.36± 3.66
Tri-Gram			82.23± 3.8	82.3± 3.77	82.71± 3.74	82.23± 3.8
Four-gram			81.52± 3.7	82.22± 3.6	81.52± 3.7	81.65± 3.65

**TABLE 18:** The Performance of Cross-Validation for Deep Neural Networks (The PolitiFact Dataset)

DL Models	Measure Performance Methods			
	Accuracy	Precision	Recall	F1-score
Modified LSTM one layer	90.49± 2.35	90.55± 4.6	83.52± 6.75	86.4± 3.73
Modified LSTM two layers	94.21± 1.49	96.15± 2.84	88.0± 3.57	91.76± 2.15
Modified LSTM three layers	90.64± 1.38	90.96± 3.95	83.68± 4.32	86.85± 1.98
Modified GRU one layer	88.8± 2.54	87.85± 7.37	78.17± 7.76	81.85± 5.93
Modified GRU two layers	87.83± 2.87	87.45± 7.38	75.17± 7.06	80.26± 6.33
Modified GRU three layers	86.05± 1.83	82.97± 6.84	75.66± 6.06	78.32± 4.81

#### 3) Testing Results for the Politifact Dataset

In this section, we discuss the generalization performance of the six machine learning models using the unseen test dataset with matrix sizes (3000).

• As the results shown in Table [19] described the testing performance of machine learning models including DT, LR, KNN, RF, SVM, NB. The DT model using four-gram achieved the best efficiency (accuracy of 75.08%, the precision of 76.63%, recall of 75.17% and F1-score 74.16%). The KNN model using uni-gram achieved the best efficiency (accuracy of 69.22%, the precision of 70.88%, recall of 71.06% and F1-score 67.59%). the RF model using bi-gram achieved the best efficiency (accuracy of 81.05%, the precision of 81.8%, recall of 81.05% and F1-score 80.71%). the LR model using tri-gram achieved the best efficiency (accuracy of 79.0%, precision of 79.29%, recall of 79.4 and F1-score 79.0%). the SVM model using Bi-gram achieved the best efficiency (accuracy of 79.06%, the precision of 79.8%, recall of 79.06% and F1-score 78.65%). The NB model using uni-gram achieved the best efficiency (accuracy of 80.42%, the precision of 80.68%, recall of 80.42% and F1-score 80.46%).
• Regarding deep learning models, as shown in [20]: The Modified LSTM, two layers achieved the best efficiency (accuracy of 83.93%, the precision of 86.66%, recall of 83.93% and F1-score 83.31%). The Modified GRU one layer achieved the best efficiency (accuracy of 81.83%, the precision of 84.53%, recall of 81.83% and F1-score 81.11%).

**TABLE 19:** The Performance of ML for Testing Result (The PolitiFact Dataset)

Feature extraction method	ML Models	Matrix Size	Measure Performance Methods			
			Accuracy	Precision	Recall	F1-score
Unigram	DT	3000	63.04	66.59	63.04	62.39
Bi-Gram			72.3	73.38	72.3	71.35
Tri-Gram			73.88	72.95	75.08	73.88
Four-gram			75.08	76.63	75.17	74.16
Unigram	KNN	3000	69.22	70.88	71.06	67.59
Bi-Gram			66.81	67.83	66.81	65.15
Tri-Gram			67.49	64.57	69.22	67.49
Four-gram			63.72	66.52	63.72	59.96
Unigram	RF	3000	68.11	73.08	68.11	67.37
Bi-Gram			81.05	81.8	81.05	80.71
Tri-Gram			79.74	79.47	80.12	79.74
Four-gram			79.63	80.18	79.63	79.32
Unigram	LR	3000	76.81	76.85	76.81	76.64
Bi-Gram			78.48	78.47	78.48	78.32
Tri-Gram			79	79.29	79.4	79
Four-gram			78.69	78.71	78.69	78.42
Unigram	SVM	3000	78.01	78.43	78.01	77.68
Bi-Gram			79.06	79.8	79.06	78.65
Tri-Gram			78.06	77.58	78.9	78.06
Four-gram			77.43	79.09	77.43	76.67
Unigram	NB	3000	80.42	80.68	80.42	80.46
Bi-Gram			78.11	79.4	78.11	78.13
Tri-Gram			76.91	76.89	78.71	76.91
Four-gram			75.5	77.73	75.5	75.42

#### 4) Experiment IV (Gossip Cop Dataset (Checking the Hollywood and Celebrity News))

[46]

#### 5) Hyperparameter Tuning

The best values parameters for two deep learning models (Modified LSTM, Modified GRU) are shown in Table [21].

**TABLE 20:** The Performance of Testing Results of Deep Neural Networks (The PolitiFact Dataset)

DL Models	Measure Performance Methods			
	Accuracy	Precision	Recall	F1-score
Modified LSTM one layer	80.26	83.54	80.26	79.33
Modified LSTM two layers	83.93	86.66	83.93	83.31
Modified LSTM three layers	82.57	84.77	82.57	81.98
Modified GRU one layer	81.83	84.53	81.83	81.11
Modified GRU two layers	79.9	82.61	79.9	78.98
Modified GRU three layers	77.17	78.74	77.17	76.42

**TABLE 21:** The Best Values Hyperparameter of Gossip Cop Dataset for Modified LSTM and Modified GRU

Models	Neurons	Dropout	reg_rate
Modified LSTM one layer	150	0.3	0.01
Modified LSTM two layers	[290,90]	[0.4,0.3]	[0.1,0.4]
Modified LSTM three layers	[490, 170, 90]	[0.3,0.9,0.4]	[0.3,0.4,0.01]
Modified GRU one layer	450	0.3	0.5
Modified GRU two layers	[250,190]	[0.2,0.4]	[0.1,0.01]
Modified GRU three layers	[270, 410, 450]	[0.9,0.7,0.6]	[0.01,0.4,0.3]

#### 6) Cross-Validation Results for Gossip Cop Dataset

In this section, the results of applying six machine learning models (D.T., L.R., KNN, RF, SVM, NB) and Two deep learning models (The Modified LSTM, The Modified GRU), including cross-validation results are described. Each machine learning model performance is discussed using four sizes of TF-IDF feature extraction, including uni-gram, bi-gram, tri-gram, four-gram, and one matrix size; 3000. In this section, the experimental evaluation of the proposed models is presented, starting by describing the experiment setup. We experimentally demonstrate the performance of the 10-fold CV results of the six machine learning models over the Gossip Cop Dataset.

• As the results shown in Table [22] described the Cross-Validation performance of machine learning models including DT, LR, KNN, RF, SVM, NB, respectively on the third dataset, the DT model using four-gram achieved the best efficiency (accuracy of 74.94%, the precision of 75.06%, recall of 74.96% and F1-score 74.76%). The KNN model using uni-gram achieved the best efficiency (accuracy of 77.62%, the precision of 77.82%, recall of 77.62% and F1-score 77.37%). The RF model using four-gram achieved the best efficiency (accuracy of 77.58%, the precision of 77.66%, recall of 77.57% and F1-score 77.41%). The LR model using four-gram achieved the best efficiency (accuracy of 79.17%, the precision of 79.32%, recall of 79.17% and F1-score 79.01%). The SVM model using uni-gram achieved the best efficiency (accuracy of 79.65%, the precision of 79.9%, recall of 79.65% and F1-score 79.42%). The NB model using uni-gram achieved the best efficiency (accuracy of 79.1%, the precision of 79.17%, recall of 79.1% and F1-score 79.03%).
• Regarding deep learning models, as shown in Table [23]: The Modified LSTM, one layer achieved the best efficiency (accuracy of 82.5%, the precision of 81.52%, recall of 79.37% and F1-score 80.0%). The Modified GRU two-layer achieved the best efficiency (accuracy of 86.05%, the precision of 82.97%, recall of 75.66% and F1-score 78.32%).

**TABLE 22:** The Performance of ML for Cross-Validation Result (Gossip Cop Dataset)

Feature extraction method	ML Models	Matrix Size	Measure Performance Methods			
			Accuracy	Precision	Recall	F1-score
Unigram	DT	3000	74.5± 1.4	74.55± 1.41	74.54± 1.42	74.28± 1.33
Bi-Gram			74.68± 1.15	74.62± 1.18	74.69± 1.15	74.47± 1.19
Tri-Gram			74.73± 1.1	74.83± 1.23	74.68± 1.22	74.52± 1.12
Four-gram			74.94± 1.32	75.06± 1.24	74.96± 1.19	74.76± 1.21
Unigram	KNN	3000	77.62± 1.35	77.82± 1.32	77.62± 1.35	77.37± 1.42
Bi-Gram			76.97± 1.26	77.21± 1.18	76.97± 1.26	76.68± 1.37
Tri-Gram			76.61± 1.29	77.14± 1.15	76.61± 1.29	76.15± 1.44
Four-gram			76.45± 1.3	76.99± 1.2	76.45± 1.3	75.99± 1.44
Unigram	RF	3000	77.49± 1.18	77.54± 1.11	77.45± 1.19	77.34± 1.16
Bi-Gram			77.52± 1.13	77.55± 1.22	77.49± 1.15	77.33± 1.15
Tri-Gram			77.58± 1.23	77.52± 1.21	77.57± 1.15	77.39± 1.2
Four-gram			77.58± 1.21	77.66± 1.21	77.57± 1.28	77.41± 1.24
Unigram	LR	3000	78.97± 1.21	79.17± 1.26	78.97± 1.21	78.74± 1.22
Bi-Gram			79.12± 1.19	79.27± 1.23	79.12± 1.19	78.92± 1.2
Tri-Gram			79.11± 1.21	79.12± 1.23	79.11± 1.21	78.97± 1.24
Four-gram			79.17± 1.22	79.32± 1.26	79.17± 1.22	79.01± 1.22
Unigram	SVM	3000	79.65± 1.2	79.9± 1.24	79.65± 1.2	79.42± 1.21
Bi-Gram			79.54± 1.19	79.66± 1.21	79.54± 1.19	79.36± 1.2
Tri-Gram			79.44± 1.16	79.55± 1.18	79.44± 1.16	79.26± 1.18
Four-gram			79.53± 1.2	79.65± 1.23	79.53± 1.2	79.35± 1.22
Unigram	NB	3000	79.1± 1.26	79.17± 1.26	79.1± 1.26	79.03± 1.26
Bi-Gram			78.97± 1.21	79.08± 1.27	78.97± 1.21	78.74± 1.22
Tri-Gram			78.98± 1.23	78.98± 1.24	78.98± 1.23	78.89± 1.24
Four-gram			78.95± 1.22	78.95± 1.24	78.95± 1.22	78.84± 1.23

**TABLE 23:** The Performance of Cross-Validation for Deep Neural Networks (Gossip Cop Dataset)

DL models	Measure Performance Methods			
	Accuracy	Precision	Recall	F1-score
Modified LSTM one layer	82.5± 1.27	81.52± 4.12	79.37± 5.84	80.0± 1.74
Modified LSTM two layers	75.18± 0.86	74.78± 2.79	68.09± 5.16	70.92± 1.88
Modified LSTM three layers	75.22± 0.69	74.32± 2.59	68.78± 5.01	71.07± 1.77
Modified GRU one layer	74.81± 0.87	73.45± 2.93	69.66± 6.6	70.98± 2.49
Modified GRU two layers	86.05± 1.83	82.97± 6.84	75.66± 6.06	78.32± 4.81
Modified GRU three layers	79.74± 0.95	78.68± 3.63	75.95± 5.28	76.88± 1.43

### E. Testing Result for the Gossip Cop Dataset

In this section, we discuss the generalization performance of the six machine learning models using the unseen test dataset with matrix sizes (3000).

• As the results show in Table [24] described the testing performance of machine learning models including DT, LR, KNN, RF, SVM, NB, respectively, the DT model using Uni-gram achieved the best efficiency (accuracy of 71.74%, the precision of 72.05%, recall of 71.74% and F1-score 71.74%). The KNN model using Tri-gram achieved the best efficiency (accuracy of 71.2%, the precision of 73.1%, recall of 71.2% and F1-score of 70.6%). The RF model using Uni-gram achieved the best efficiency (accuracy of 76.2%, the precision of 76.91%, recall of 76.2% and F1-score 76.04%). The LR model using Uni-gram achieved the best efficiency (accuracy of 76.2%, precision 76.63%, recall of 76.2%, and F1-score 76.1%). The SVM model using uni-gram achieved the best efficiency (accuracy of 77.51%, precision 78.11%, recall of 77.51%, and F1-score 77.39%). The NB model using uni-gram achieved the best efficiency (accuracy of 76.2%, precision 76.63%, recall of 76.2%, and F1-score 76.1%).
• Regarding deep learning models, as shown in Table [25]: The Modified LSTM, one layer achieved the best efficiency (accuracy of 83.82%, precision 84.85%, recall of 83.82%, and F1-score of 83.7%). The Modified GRU two layers achieved the best efficiency (accuracy of 81.49%, precision 82.26%, recall of 81.49, and F1-score 81.35%).

**TABLE 24:** The Performance of ML for Testing Result (Gossip Cop Dataset)

Feature extraction method	ML Models	Matrix Size	Measure Performance Methods			
			Accuracy	Precision	Recall	F1-score
Unigram	DT	3000	71.74	72.05	71.74	71.64
Bi-Gram			71.6	71.94	71.6	71.48
Tri-Gram			71.51	71.9	71.51	71.38
Four-gram			71.17	71.57	71.17	71.03
Unigram	KNN	3000	71.41	72.43	71.41	71.09
Bi-Gram			70.61	72.79	70.61	69.89
Tri-Gram			71.2	73.1	71.2	70.6
Four-gram			70.74	72.89	70.74	70.04
Unigram	RF	3000	76.2	76.91	76.2	76.04
Bi-Gram			75.52	76.26	75.52	75.35
Tri-Gram			75.36	76.02	75.36	75.21
Four-gram			75.28	75.9	75.28	75.14
Unigram	LR	3000	76.2	76.63	76.2	76.1
Bi-Gram			76.12	76.51	76.12	76.03
Tri-Gram			75.88	76.0	75.88	75.85
Four-gram			75.94	76.29	75.94	75.86
Unigram	SVM	3000	77.51	78.11	77.51	77.39
Bi-Gram			76.56	77.04	76.56	76.46
Tri-Gram			76.34	76.76	76.34	76.25
Four-gram			76.29	76.68	76.29	76.2
Unigram	NB	3000	76.2	76.63	76.2	76.1
Bi-Gram			75.62	75.72	75.62	75.59
Tri-Gram			75.35	75.41	75.35	75.33
Four-gram			75.48	75.53	75.48	75.47

**TABLE 25:** The Performance of ML for Testing Results (Gossip Cop Dataset)

	Accuracy	Precision	Recall	F1-score	
Modified LSTM one layer	83.82	84.85	83.82	83.7	
Modified LSTM two layers	75.77	76.64	75.77	75.55	
Modified LSTM three layers	76.67	77.33	76.67	76.52	
Modified GRU one layer	77.65	78.03	77.65	77.57	
Modified GRU two layers	81.49	82.26	81.49	81.35	
Modified GRU three layers	81.04	81.37	81.04	80.99	

## V. Discussion

### A. Discussion for Experiment I(COVID-19 Dataset)

**FIGURE 7.**
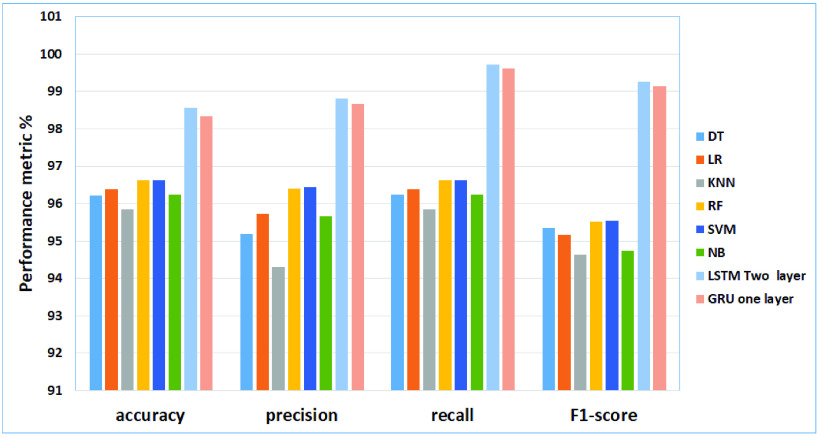
The cross-validation performances results for the COVID-19 dataset.

**FIGURE 8.**
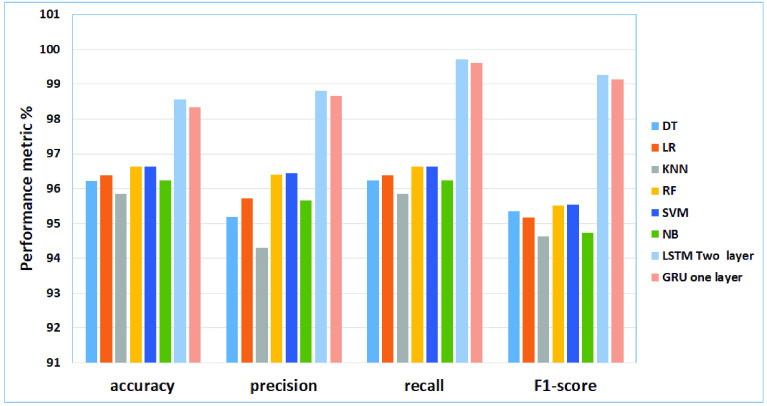
The testing results for the COVID-19 dataset.

From the results obtained in our experiments for the COVID-19 dataset, **Figure 7** and **Figure 8** depict the empirical results in the big picture for the cross-validation performances and the testing results, respectively. They are showing the performance of the best models for each feature extraction method. To summarize the performance of the compared models, we explore the average cross-validation and the testing results of each model using different sizes of feature extraction methods, N-gram from n $= {1}$ to n = 4, and the matrix is 3000 for baseline machine learning (DT, LR, KNN, RF, SVM, NB). The feature is extracted using the word embedding method for Deep Neural Network (The Modified LSTM, and The Modified GRU). On average, The Modified LSTM model has obtained the best cross-validation average and in the testing performance average compared to other regular machine learning models. For cross-validation results and testing results, the Modified LSTM (Two layers) model has obtained the best an accuracy. for cross validation results; Accuracy 98.57%, precision of 98.82%, recall of 99.71%, and F1-score of 99.26%. For performance testing, The Modified LSTM has achieved an accuracy of 98.6%, precision of 98.55%, recall of 98.6%, and F1-score of 98.5%. The Modified GRU has obtained the second-best rank of cross-validation and testing performance using one layer. for cross validation results; (accuracy of 98.33%, the precision of 98.67%, recall of 99.62%, and F1-score of 99.14%). Also, it has been reported to be the second-best results for testing (accuracy of 98.29%, the precision of 98.24%, recall of 98.29%, and F1-score of 98.21%).

SVM has obtained the third-best result on the average of cross-validation performance, and in the testing results. For cross-validation results, SVM using Bi-gram has recorded achieved (accuracy of 96.64%, precision 96.45%, recall of 96.64%, and F1-score 95.53%). For testing results, SVM has recorded using Uni-gram (accuracy of 96.38%, the precision of 96.18%, recall of 96.38% and F1-score 95.05%). RF has obtained the fourth-best result on the average of cross-validation performance and the testing results. For cross-validation results, it has recorded using Bi-gram (accuracy of 96.63%, the precision of 96.41%, recall of 96.63% and F1-score 95.52%). For testing results, it has recorded using Bi-gram (accuracy of 96.35%, the precision of 96.07%, recall of 96.35% and F1-score 95.0%). NB has obtained the fifth-best result on the average of cross-validation performance, and in the testing results. For cross-validation results, NB has recorded achieved using Four-gram (accuracy of 96.24%, the precision of 95.67%, recall of 96.24% and F1-score 94.73%). For testing results, using Tri-gram obtained (accuracy of 96.13%, the precision of 95.74%, recall of 96.13%, and F1-score 94.5%). LR has obtained the sixth-best result on the average of cross-validation performance and the testing results. For cross-validation results, it has recorded using Bi-gram (accuracy of 96.38%, the precision of 95.73%, recall of 96.38% and F1-score 95.17%)). For testing results, it has recorded using Four-gram (accuracy of 95.82%, the precision of 94.24%, recall of 95.82%, and F1-score 94.59%). DT and KNN have reported the lowest cross-validation and testing results. DT achieved the seventh rank on the average of cross-validation performance, and KNN achieved the best in the testing results. For cross-validation results, DT has recorded achieved using uni-gram (accuracy of 96.22%, precision 95.19%, recall of 96.23% and F1-score 95.35%). For testing results, KNN has recorded using uni-gram achieved the best efficiency (accuracy of 95.66%, precision 92.16%, recall of 95.66% and F1-score 93.81%). KNN achieved the last rank on the average of cross-validation performance, and DT achieved the best in the testing results. For cross-validation results, KNN has recorded achieved using Four-gram (accuracy of 95.87%, the precision of 94.35%, recall of 95.87% and F1-score 94.66%).; and for testing results, DT has recorded using uni-gram (accuracy of 95.0%, the precision of 93.71%, recall of 95.0 % and F1-score 94.26%)

Consequently, The Modified LSTM and The Modified GRU for the COVID-19 dataset are outperforming SVM, DT, RF, NB, LR, and KNN for cross-validation and testing results. Based on these results, it can be tentatively concluded that The Modified LSTM and The Modified GRU classifiers will be used in the fake news detection model.

### B. Discussion for Experiment II (Disasters Dataset)

**FIGURE 9.**
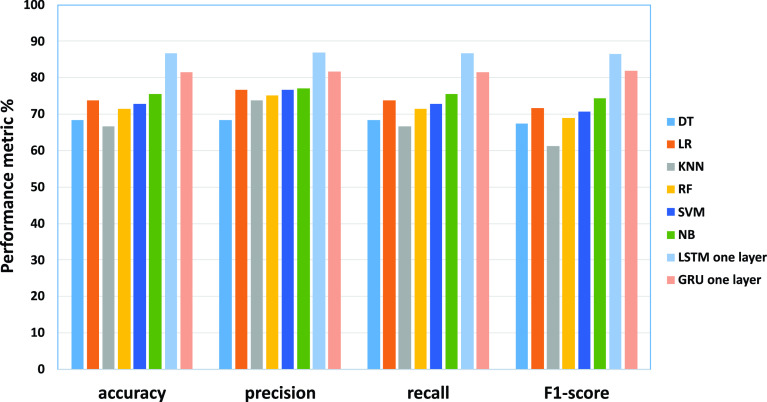
The cross-validation performances results for the disasters dataset.

**FIGURE 10.**
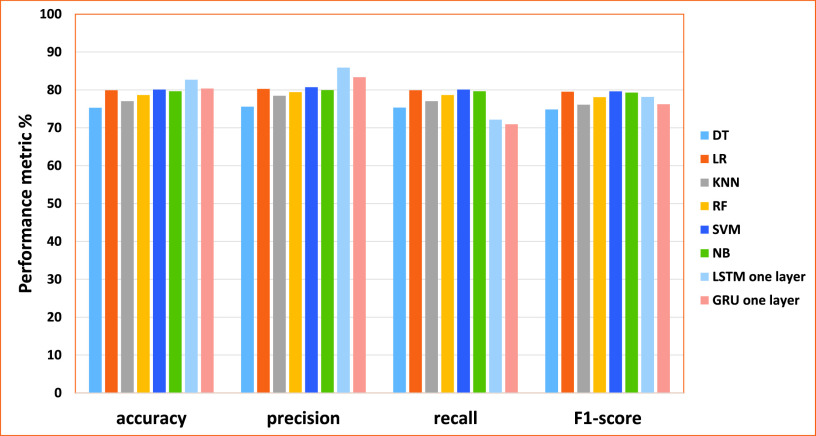
The testing results for the disasters dataset.

From the results obtained in our experiments for the disasters dataset, **Figure 9** and **Figure 10** depict the empirical results in the big picture for the cross-validation performances and the testing results, respectively. On average, The Modified LSTM (one layer) model has obtained the best cross-validation average and the testing performance average compared to other regular machine learning models. For cross-validation results, the Modified LSTM model has achieved an accuracy of 82.68%, precision of 85.86%, recall of 72.13%, and F1-score of 78.14% using one layer. For performance testing, The Modified LSTM has achieved an accuracy of 86.74%, precision of 86.98%, recall of 86.74%, and F1-score of 86.6% using also one layer. The Modified GRU has obtained the second-best rank of cross-validation performance using one layer (accuracy of 80.37%, the precision of 83.37%, recall of 70.93%, and F1-score of 76.21%).

Also, it has been reported to be the second-best results using two layers of performance testing (accuracy of 81.44%, the precision of 81.68%, recall of 81.44%, and F1-score of 81.81%).

SVM has obtained the third-best result on the average of cross-validation performance, and NB achieved the best in the testing results. For cross-validation results, SVM using Bi-gram has recorded achieved (accuracy of 80.08%, precision 80.69%, recall of 80.08%, and F1-score 79.61%). For testing results, NB has recorded using Four-gram (accuracy of 76.2%, the precision of 76.26%, recall of 76.2% and F1-score 75.81%).

LR has obtained the fourth-best result on the average of cross-validation performance and the testing results. For cross-validation results, it has recorded using Bi-gram (accuracy of 79.91%, the precision of 80.28%, recall of 79.91% and F1-score 79.53%). For testing results, it has recorded using Four-gram (accuracy of 73.7%, the precision of 76.65%, recall of 73.7% and F1-score 71.77%).’ NB has obtained the fifth-best result on the average of cross-validation performance, and SVM achieved the best in the testing results. For cross-validation results, NB has recorded achieved using uni-gram (accuracy of 79.65%, the precision of 79.96%, recall of 79.65% and F1-score 79.28%). For testing results, SVM has recorded using Bi-gram obtained (accuracy of 72.9%, the precision of 76.65%, recall of 72.9%, and F1-score 70.6%).

RF has obtained the sixth-best result on the average of cross-validation performance and the testing results. For cross-validation results, it has recorded using uni-gram (accuracy of 78.64%, the precision of 79.41%, recall of 78.64% and F1-score 78.08%)). For testing results, it has recorded using uni-gram (accuracy of 71.48%, the precision of 75.08%, recall of 71.48%, and F1-score 69.04%).

DT and KNN have reported the lowest cross-validation and testing results.KNN achieved the seventh rank on the average of cross-validation performance, and DT achieved the seventh in the testing results. For cross-validation results, KNN has recorded achieved using uni-gram (accuracy of 77.03%, precision 78.44%, recall of 77.03% and F1-score 76.08%). For testing results, DT has recorded using uni-gram achieved the best efficiency (accuracy of 68.42%, precision 68.38%, recall of 68.42% and F1-score 67.44%). DT achieved the last rank on the average of cross-validation performance, and KNN achieved the best in the testing results. For cross-validation results, DT has recorded achieved using uni-gram (accuracy of 75.3%, the precision of 75.58%, recall of 75.31% and F1-score 74.83%).; and for testing results, KNN has recorded using uni-gram (accuracy of 66.68%, the precision of 73.85%, recall of 66.68 % and F1-score 61.16%).

Consequently, The Modified LSTM and The Modified GRU for the disasters dataset are outperforming SVM, DT, RF, NB, LR, and KNN for cross-validation and testing results. Based on these results, it can be tentatively concluded that The Modified LSTM and The Modified GRU classifiers will be used in the fake news detection model.

### C. Discussion for Experiment III (Politifact Dataset)

**FIGURE 11.**
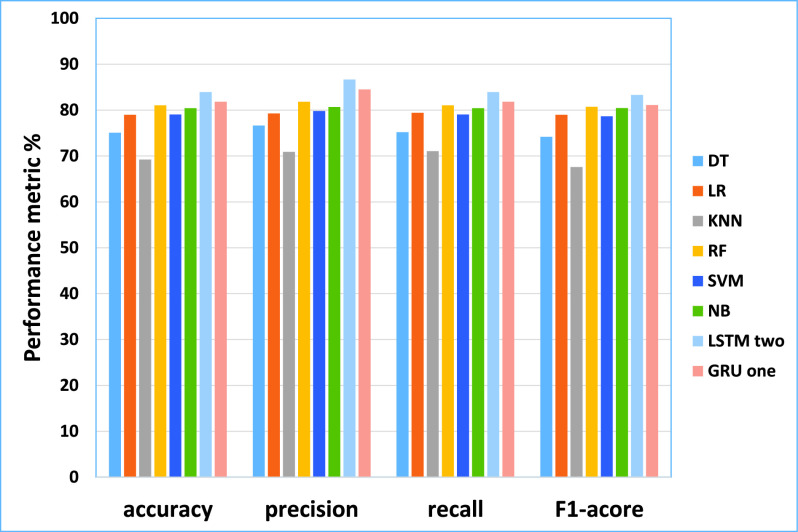
The cross-validation performances results for the PolitiFact dataset.

**FIGURE 12.**
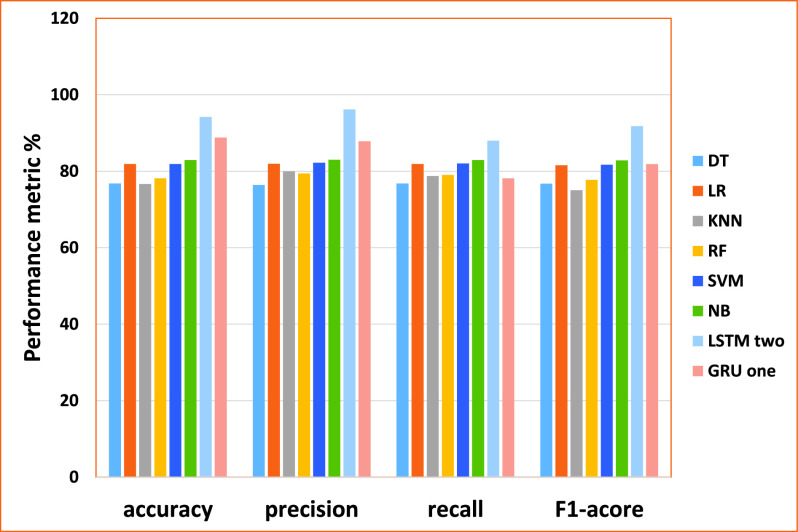
The testing results for the PolitiFact dataset.

From the results obtained in our experiments for the PolitiFact data set, **Figure 11** and **Figure 12** depict the empirical results in the big picture for the cross-validation performances and the testing results, respectively. On average, The Modified LSTM (Two-layer) model has obtained the best cross-validation average and the testing performance average compared to other regular machine learning models.

For cross-validation results, the Modified LSTM model has achieved an accuracy of 94.21%, precision of 96.15%, recall of 88.0%, and F1-score 91.76%). For performance testing, The Modified LSTM has achieved an accuracy of 83.93%, precision of 86.66%, recall of 83.93%, and F1-score 83.31%). The Modified GRU (One Layer) has obtained the second-best rank of cross-validation performance using one layer (accuracy of 88.8%, precision 87.85%, recall of 78.17%, and F1-score 81.85%). Also, it has been reported to be the second-best results of performance testing (accuracy of 81.83%, precision 84.53%, recall of 81.83% and F1-score 81.11%).

NB has obtained the third-best result on the average of cross-validation performance, and RF achieved the best in the testing results.

For cross-validation results, NB using Uni-gram has recorded achieved (accuracy of 82.92%, precision 83.01%, recall of 82.92% and F1-score 82.82%). For testing results, RF has recorded using Bi-gram (accuracy of 81.05%, precision 81.8%, recall of 81.05%, and F1-score 80.71%). LR has obtained the fourth-best result on the average of cross-validation performance, and NB achieved the best in the testing results. For cross-validation results, LR using Bi-gram has recorded achieved (accuracy of 81.91%, the precision 82.23%, recall of 82.02% and F1-score 81.69%). For testing results, NB has recorded using the Uni-gram (accuracy of 80.42%, the precision of 80.68%, recall of 80.42%, and F1-score 80.46%). LR has obtained the fifth-best result on the average of cross-validation performance, and SVM achieved the best in the testing results. For cross-validation results, LR has recorded achieved using Tri-gram (accuracy of 81.91%, precision 81.94%, recall of 81.91% and F1-score 81.55%). For testing results, SVM has recorded using Bi-gram obtained (accuracy of 79.06%, precision 79.8%, recall of 79.06%, and F1-score 78.65%). RF has obtained the sixth-best result on the average of cross-validation performance, and LR achieved the best in the testing results. For cross-validation results, RF has recorded achieved using Bi-gram (accuracy of 78.15%, precision, 79.42%, recall of 79.02% and F1-score 77.74%). For testing results, LR has recorded using Tri-gram obtained (accuracy of 79.0%, precision 79.29%, recall of 79.4% and F1-score 79.0%).

DT and KNN have reported the lowest cross-validation and testing results.KNN achieved the seventh rank on the average of cross-validation performance, and DT achieved the best in the testing results. For cross-validation results, KNN has recorded achieved using uni-gram (accuracy of 76.67%, precision 79.96%, recall of 78.72% and F1-score 75.02%). For testing results, DT has recorded using Four-gram achieved the best efficiency (accuracy of 75.08%, precision 76.63%, recall of 75.17% and F1-score 74.16%). DT achieved the last rank on the average of cross-validation performance, and KNN achieved the best in the testing results. For cross-validation results, DT has recorded achieved using Four-gram (accuracy of 76.8%, precision 76.41%, recall of 76.8%, and F1-score 76.73%). For testing results, KNN has recorded using uni-gram (accuracy of 69.22%, precision 70.88%, recall of 71.06%, and F1-score 67.59%).

Consequently, The Modified LSTM and The Modified GRU for the PolitiFact dataset are outperforming SVM, DT, RF, NB, LR, and KNN for cross-validation and testing results. Based on these results, it can be tentatively concluded that The Modified LSTM and The Modified GRU classifiers will be used in the fake news detection model.

### D. Discussion for Case Study IV (Gossip Cop Dataset)

**FIGURE 13.**
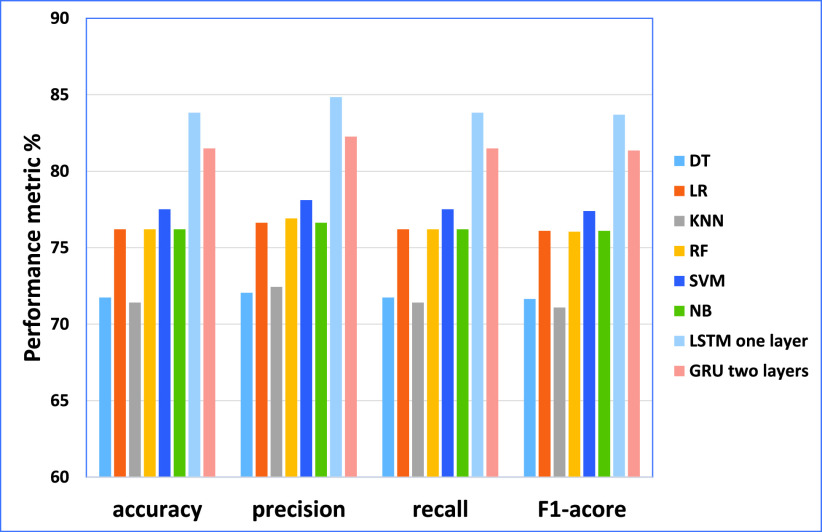
The cross-validation performances results for the gossip cop dataset.

**FIGURE 14.**
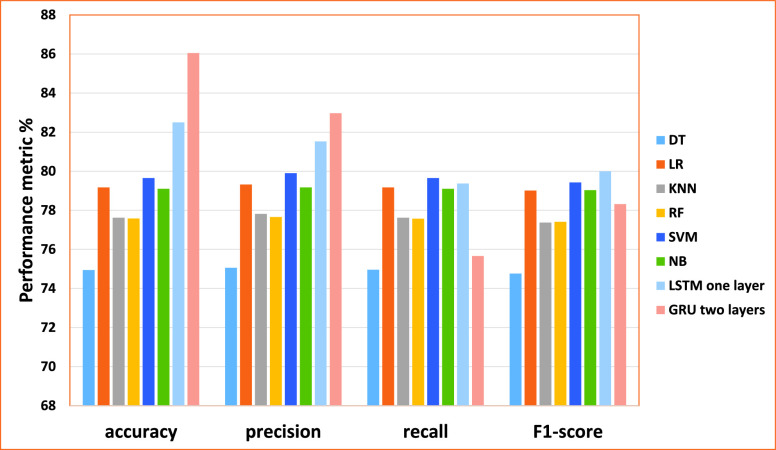
The testing results for the gossip cop dataset.

From the results obtained in our experiments for the gossip cop dataset, **figures 13** and **14** depict the empirical results in the big picture for the cross-validation performances and the testing results, respectively. On average, The Modified GRU (Two-layer) model has obtained the best cross-validation average, and The Modified LSTM (one layer) has obtained the best testing performance average compared to other regular machine learning models. For cross-validation results, The Modified GRU (two layers) model has achieved (accuracy of 86.05%, precision 82.97%, recall of 75.66% and F1-score 78.32%). For performance testing, The Modified LSTM (one layer) has achieved (accuracy of 83.82%, precision 84.85%, recall of 83.82%, and F1-score 83.7%). The Modified LSTM (One Layer) has obtained the second-best rank of cross-validation performance(accuracy of 82.5%, precision 81.52%, recall of 79.37%, and F1-score 80.0%). Also, The Modified GRU (Two-layer) has been reported to be the second-best testing results using (accuracy of 81.49%, precision 82.26%, recall of 81.49%, and F1-score 81.35%).

SVM (Uni-Gram) has obtained the third-best result on the average of cross-validation performance and testing results. For cross-validation results, it recorded (accuracy of 79.65%, the precision of 79.9%, recall of 79.51%, and F1-score 79.42%). Furthermore, For testing results, it recorded (accuracy of 77.51%, the precision 78.11%, recall of 77.51%, and F1-score 77.39%).

LR (Four Gram) has obtained the fourth-best result on the average of cross-validation performance, and RF (Uni Gram) achieved the best in the testing results. For cross-validation results, LR using Four-gram has recorded achieved (accuracy of 79.17%, precision 79.32%, recall of 79.17% and F1-score 79.01%). For testing results, RF has recorded using Uni-gram (accuracy of 76.2%, precision 76.91%, recall of 76.2%, and F1-score 76.04%). NB has obtained the fifth-best result on the average of cross-validation performance, and LR achieved the best in the testing results. For cross-validation results, NB has recorded achieved using Uni-gram (accuracy of 79.1%, precision 79.17%, recall of 79.1%, and F1-score 79.03%). For testing results, LR has recorded using Uni-gram obtained (accuracy of 76.2%, precision 76.63%, recall of 76.2%, and F1-score 76.1 %). KNN has obtained the sixth-best result on the average of cross-validation performance, and NB achieved the best in the testing results. For cross-validation results, KNN has recorded achieved using Uni-gram (accuracy of 77.62%, precision of 77.82%, recall of 77.62% and F1-score 77.37%). For testing results, NB has recorded using Uni-gram obtained (accuracy of 76.2%, precision 76.63%, recall of 76.2%, and F1-score 76.1%). RF achieved the Seventh rank on the average of cross-validation performance, and DT achieved the best in the testing results. For cross-validation results, RF has recorded achieved using Four-gram (accuracy of 77.58%, precision of 77.66%, recall of 77.57%, and F1-score 77.41%). For testing results, DT has recorded using Uni-gram obtained (accuracy of 71.74%, precision 72.05%, recall of 71.74%, and F1-score 71.64%). DT and KNN have reported the lowest cross-validation and testing results respectively. DT (Four-Gram) achieved the last rank on the average of cross-validation performance, and KNN achieved the best in the testing results. For cross-validation results, DT has recorded achieved using four-gram (accuracy of 74.94%, precision 75.06%, recall of 74.96% and F1-score 74.76%). For testing results, KNN has recorded using Tri-gram achieved the best efficiency (accuracy of 71.2%, precision 73.1%, recall of 71.2% and F1-score 70.6%).

Consequently, The Modified LSTM and The Modified GRU for the gossip cop dataset are outperforming SVM, DT, RF, NB, LR, and KNN for cross-validation and testing results. Based on these results, it can be tentatively concluded that The Modified GRU and The Modified LSTM classifiers will be used in the fake news detection model

## VI. Conclusion and Future Work

One of the most threatening events is the spread of COVID-19 virus. People seek trustworthy information from social media to learn how to protect themselves. Misinformation can kill people. In this paper, we proposed efficient and enhanced deep learning techniques to detect fake news from COVID-19 dataset and three other datasets (disasters, politifact and gossip cop). All experiments are packed up by cross validation and testing on unseen data to support the validity of our models. Regarding COVID-19 dataset, the best testing results are obtained by **LSTM (two layers)**. The performance measure results are as follows: the accuracy is 98.6%, the precision is 98.55%, the recall is 98.6% and F1-score is 98.5%. Regarding disasters dataset, the best testing results are obtained by **The Modified LSTM (one layer)**. The performance measure results are as follows: the accuracy is 86.74%, the precision is 86.98%, the recall is 86.74% and F1-score is 86.6%. Regarding politifact dataset, the best testing results are obtained by **The Modified LSTM (two layers)**. The performance measure results are as follows: the accuracy is 83.93%, the precision is 86.66%, the recall is 83.93% and F1-score is 83.31%. Regarding gossip cop dataset, the best testing results are obtained by **The Modified LSTM (one layer)**. The performance measure results are as follows: the accuracy is 83.82, the precision is 84.85%, the recall is 83.82% and F1-score is 83.7%. We can conclude that the Modified LSTM with tuned parameters (with one or two layers) proposed in this paper outperformed DT, KNN, RF, LR, SVM, NB and baseline The Modified LSTM and The Modified GRU models. The main strength in our proposed approaches is the preprocessing stage which depends on word embedding. Moreover, Some parameters such as the number of neurons in each layer and the drop out ratio greatly affect on the performance of the deep learning technique. These parameters are optimized to obtain the best performance. In this paper, the content features are used in the binary classification. In the future, we intend to use a combination of content, temporal, and context features to be used in multi-class classification. Capsule networks can be included in future plans to detect their effect on the performance.

In this paper, we proposed optimized machine learning and deep learning systems to detect fake news for COVID 19 and other data-sets. The prepossessing stage contained an elaborate sentence analysis starting from removing unimportant characters till tokenization and stemming. Three different datasets are used and split into training and testing sections. The feature analysis of machine learning approach depends on TF-IDF and Ngrams, while the deep learning approach depends on word embedding. Both approaches are optimized using grid search and Keras tuning, respectively. The performance of both approaches is measured using accuracy, precision, recall, and F1-measure. The deep learning approaches outperformed the machine learning approaches in the three datasets. However, The Modified LSTM (two layers) achieved the highest cross-validation accuracy (94.21%) using the second dataset. While The Modified LSTM (one layer) achieved the highest testing accuracy (86.74 %) using the first dataset. We recommend the Keras tuned The Modified LSTM approach as a deep learning approach for fake news detection.

## Conflicts of Interest

The authors have declared that there is no conflict of interest. Non-financial competing interests.

## Author Contributions

All authors contributed equally to this paper, where Diaa Salama participated in sorting the experiments, discussed and analyzed the results, performed the experiments and analyzed the results, wrote the paper, discussed the result, and revised/edited the manuscript. Mohamed Taha, and Ahmed Taha: performed the experiments and analyzed the results s and wrote the paper. Fatma Helmy: discussed the results and wrote the paper. Ayma Nabil: discussed the results and revised the paper. Essam H Houssein: analyzed the results and revised the paper. All authors reads and approved the work in this paper.
